# Synthesis and Evaluation of ^68^Ga- and ^177^Lu-Labeled [diF-Pro^14^]Bombesin(6−14) Analogs for Detection and Radioligand Therapy of Gastrin-Releasing Peptide Receptor-Expressing Cancer

**DOI:** 10.3390/ph18020234

**Published:** 2025-02-08

**Authors:** Lei Wang, Chao-Cheng Chen, Devon Chapple, Antonio A. W. L. Wong, Sara Kurkowska, Wing Sum Lau, Carlos F. Uribe, François Bénard, Kuo-Shyan Lin

**Affiliations:** 1 Department of Molecular Oncology, BC Cancer Research Institute, Vancouver, BC V5Z 1L3, Canada; lewang@bccrc.ca (L.W.); ccchen@bccrc.ca (C.-C.C.); devchapple@gmail.com (D.C.); antwong@bccrc.ca (A.A.W.L.W.); wlau@bccrc.ca (W.S.L.); fbenard@bccrc.ca (F.B.); 2 Department of Integrative Oncology, BC Cancer Research Institute, Vancouver, BC V5Z 1L3, Canada; skurkowska@bccrc.ca (S.K.); curibe@bccrc.ca (C.F.U.); 3 Department of Nuclear Medicine, Pomeranian Medical University, 70-204 Szczecin, Poland; 4 Department of Molecular Imaging and Therapy, BC Cancer, Vancouver, BC V5Z 4E6, Canada; 5 Department of Radiology, University of British Columbia, Vancouver, BC V5Z 1M9, Canada

**Keywords:** gastrin-releasing peptide receptor, 4,4-difluoroproline, gallium-68, lutetium-177, pancreas uptake

## Abstract

**Background/Objectives:** Overexpressed in various solid tumors, the gastrin-releasing peptide receptor (GRPR) is a promising target for cancer diagnosis and therapy. However, the high pancreas uptake of the current clinically evaluated GRPR-targeted radiopharmaceuticals limits their applications. In this study, we replaced the Pro^14^ residue in our previously reported GRPR-targeted LW02056 and ProBOMB5 with 4,4-difluoroproline (diF-Pro) to obtain an agonist LW02060 (DOTA-Pip-[D-Phe^6^,Tle^10^,NMe-His^12^,diF-Pro^14^]Bombesin(6–14)) and an antagonist LW02080 (DOTA-Pip-[D-Phe^6^,NMe-Gly^11^,Leu^13^(ψ)diF-Pro^14^]Bombesin(6–14)), respectively. **Methods/Results**: The binding affinities (K_i_) of Ga-LW02060, Ga-LW02080, Lu-LW02060, and Lu-LW02080 were measured by in vitro competition binding assays using PC-3 cells and were found to be 5.57 ± 2.47, 21.7 ± 6.69, 8.00 ± 2.61, and 32.1 ± 8.14 nM, respectively. The ^68^Ga- and ^177^Lu-labeled ligands were obtained in 36–75% decay-corrected radiochemical yields with >95% radiochemical purity. PET imaging, SPECT imaging, and ex vivo biodistribution studies were conducted in PC-3 tumor-bearing mice. Both [^68^Ga]Ga-LW02060 and [^68^Ga]Ga-LW02080 enabled clear tumor visualization in PET images at 1 h post-injection (pi). Tumor uptake values of [^68^Ga]Ga-LW02060 and [^68^Ga]Ga-LW02080 at 1 h pi were 16.8 ± 2.70 and 7.36 ± 1.33 %ID/g, respectively, while their pancreas uptake values were 3.12 ± 0.89 and 0.38 ± 0.04 %ID/g, respectively. Compared to [^177^Lu]Lu-LW02080, [^177^Lu]Lu-LW02060 showed higher tumor uptake at all time points (1, 4, 24, 72, and 120 h pi). However, fast tumor clearance was observed for both [^177^Lu]Lu-LW02060 and [^177^Lu]Lu-LW02080. **Conclusions:** Our data demonstrate that [^68^Ga]Ga-LW02060 is promising for clinical translation for the detection of GRPR-expressing tumor lesions. However, further optimizations are needed for [^177^Lu]Lu-LW02060 and [^177^Lu]Lu-LW02080 to prolong tumor retention for therapeutic applications.

## 1. Introduction

Gastrin-releasing peptide receptor (GRPR) is a member of G-protein coupled receptors and is expressed in the human body, including in the pancreas, gastrointestinal tract, and central nervous system [1,2]. It is involved in regulating many physiological functions, such as hormone secretion, smooth muscle contraction, and synaptic plasticity [1,2]. Furthermore, GRPR is found to be significantly overexpressed in various solid malignancies, including breast, prostate, lung, and colon cancers, where it functions as a tumor growth factor [3,4,5,6,7,8]. Such characteristics of GRPR makes it an attractive target for developing targeted radiopharmaceuticals for diagnosis and radioligand therapy of GRPR-expressing cancer.

Bombesin (BBN) is a natural peptide showing a very potent binding affinity to GRPR. Its *C*-terminal heptapeptide, bombesin(8–14), has been identified as the sequence needed for binding to GRPR and is widely utilized for the design of GRPR-targeted pharmaceuticals [9,10,11,12,13,14,15,16]. However, most clinically evaluated GRPR-targeted radiopharmaceuticals derived from BBN showed extremely high pancreas uptake, which not only limits the detection of GRPR-expressing tumor lesions in and near the pancreas but also lowers the maximum tolerated dose for radiotherapeutic applications [9,10,11,12,13,14,17]. Moreover, extracellularly enzymatic degradation by neutral endopeptidase 24.11 (NEP, EC 3.4.24.11, neprilysin) results in low in vivo stability for most of the reported GRPR-targeted radiopharmaceuticals derived from the BBN sequence [18,19].

**Figure 1:**
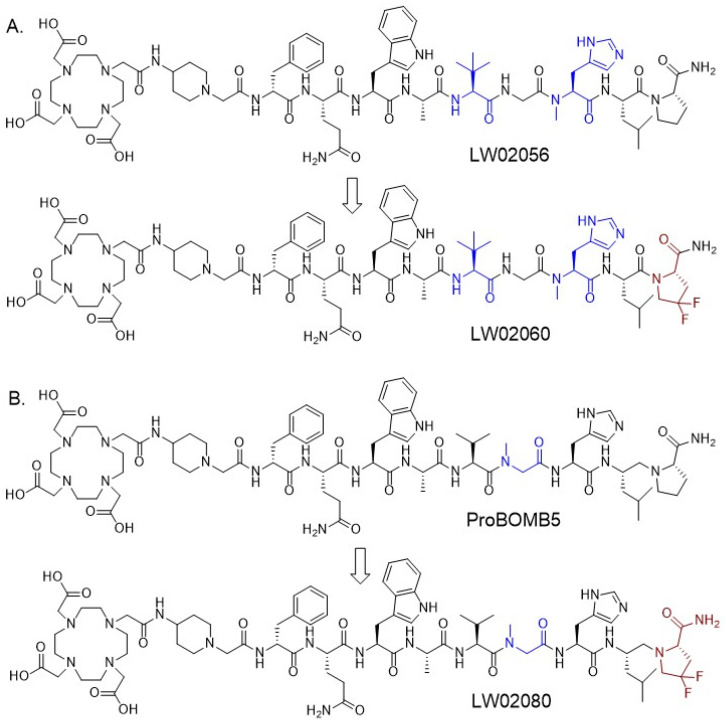
Chemical structures of (**A**) LW02056 and LW02060 and (**B**) ProBOMB5 and LW02080. Unnatural amino acid substitutions (Tle^10^, NMe-Gly^11^, and NMe-His^12^) are in blue, and diF-Pro^14^ is in brown.

To address these two major limitations, our group previously developed a series of GRPR-targeted radiopharmaceuticals [20,21]. These ligands were derived from RC-3950-II ([D-Phe^6^,Leu^13^(ψ)Thz^14^]Bombesin(6–14)), which has a Thz^14^ (thiazoline-4-carboxylic acid) substitution and a reduced peptide bond (CH_2_-N) between residues 13–14 (Leu^13^(ψ)Thz^14^) [15,16]. All the ^68^Ga-labeled GRPR antagonists derived from the [Leu^13^(ψ)Thz^14^]Bombesin(6–14) pharmacophore and all the ^68^Ga-labeled GRPR agonists derived from the [Thz^14^]Bombesin(6–14) pharmacophore showed low pancreas uptake (0.78–7.26 %ID/g at 1 h pi) [20,21]. Subsequently, our group improved the in vivo stability of the lead candidates by systematically substituting natural amino acids at potential cleavage sites (Gln^7^, Trp^8^, Ala^9^, Val^10^, Gly^11^, and His^12^) with unnatural amino acids. This led to the discoveries of several GRPR-targeted radiopharmaceuticals with significantly improved in vivo stability [22,23]. We discovered that Tle^10^ and NMe-His^12^ substitutions, either in combination or alone, can significantly enhance the in vivo stability and improve the tumor uptake of ^68^Ga-labeled GRPR agonists derived from the [Thz^14^]Bombesin(6–14) pharmacophore [22]. We also identified that NMe-Gly^11^ substitution can improve the tumor uptake and tumor-to-organ uptake ratios of ^68^Ga-labeled GRPR antagonists derived from the [Leu^13^(ψ)Thz^14^]Bombesin(6–14) pharmacophore [20,23]. Recently, we reported two novel GRPR-targeted ligands, LW02056 and ProBOMB5 (Figure 1), by replacing the Thz^14^ in our previously identified LW01110 and TacsBOMB5, respectively, with Pro^14^ to avoid oxidation of Thz in the product formulation [24]. Though a slight reduction was observed for the tumor uptake of [^68^Ga]Ga-LW02056 (8.93 ± 1.96 %ID/g at 1 h pi), it had very low pancreas uptake (1.31 ± 0.42 %ID/g at 1 h pi) and good tumor-to-background imaging contrast [24]. The Pro^14^-derived antagonist, [^68^Ga]Ga-ProBOMB5, showed good tumor uptake (12.4 ± 1.35 %ID/g), low pancreas uptake (1.37 ± 0.40 %ID/g), and excellent tumor-to-background imaging contrast at 1 h pi [24].

Fluorinated proline derivatives play a crucial role in peptide engineering and drug discovery due to their resistance to degradation by NEP and their ability to modulate polarity and lipophilicity [25,26]. A 4,4-difluoroproline (diF-Pro) derivative, 4,4-difluoropyrrolidine-2-carbonitrile, has been successfully introduced in the design of potent fibroblast activation protein (FAP) inhibitors to mimic Pro [27], and now 4,4-difluoropyrrolidine-2-carbonitrile is widely used for the design of FAP-targeted radiopharmaceuticals [28]. In this study, we synthesized two GRPR-targeted ligands, LW02060 and LW02080, by replacing Pro^14^ of LW02056 and ProBOMB5, respectively, with diF-Pro^14^ (Figure 1). Subsequently, we characterized the antagonist/agonist characteristics of these two ligands and their Ga/Lu-complexed analogs. We also assessed their potential for PET imaging and targeted radiotherapy using a preclinical tumor model derived from GRPR-expressing PC-3 prostate cancer cells. We hypothesized that (1) diF-Pro^14^ substitution would retain GRPR agonist/antagonist characteristics and good binding affinity to GRPR; (2) compared with their Pro^14^ analogs, the diF-Pro^14^-derived radioligands would have comparable or even higher in vivo stability and could be promising for the detection and radioligand therapy of GRPR-expressing malignancies.

## 2. Results

### 2.1. Chemistry and Radiochemistry

LW02060 and LW02080 were synthesized on solid phase using Fmoc chemistry, and their isolated yields were 23% and 39%, respectively. The isolated yields for the synthesis of Ga-LW02060 and Ga-LW02080 were 87% and 93%, respectively, and the isolated yields for Lu-LW02060 and Lu-LW02080 were 87% and 90%, respectively (Tables S1 and S2). ^68^Ga-labeled LW02060 and LW02080 were obtained in 41–75% decay-corrected radiochemical yields with >172 GBq/µmol molar activity and >95% radiochemical purity (Table S3). ^177^Lu-labeled LW02060 and LW02080 were obtained in 36–64% decay-corrected radiochemical yields with >125 GBq/µmol molar activity and >97% radiochemical purity (Table S3).

### 2.2. Agonist/Antagonist Characterization, Binding Affinity, and Hydrophilicity

**Figure 2:**
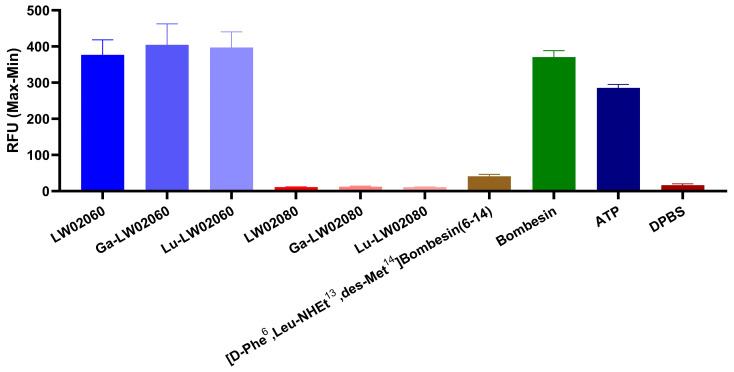
Intracellular calcium efflux in PC-3 cells. Cells were induced with 50 nM of tested ligands. Error bars indicate standard deviation (n = 4).

All LW02060 and its Ga/Lu-complexed analogs were confirmed to be GRPR agonists, while all LW02080 and its Ga/Lu-complexed analogs were confirmed to be GRPR antagonists by intracellular calcium release assays using PC-3 cells (Figure 2). A total of 50 nM of LW02060, Ga-LW02060, Lu-LW02060, ATP (positive control), and bombesin (agonist control) induced Ca^2+^ efflux corresponding to 377 ± 41.9, 405 ± 58.3, 397 ± 43.4, 286 ± 9.44, and 371 ± 17.9 RFU (relative fluorescent unit), respectively. By contrast, 50 nM of LW02080, Ga-LW02080, Lu-LW02080, [D-Phe^6^,Leu-NHEt^13^,des-Met^14^]Bombesin(6–14) (antagonist control), and Dulbecco’s phosphate-buffered saline (DPBS, blank control) induced Ca^2+^ efflux corresponding to only 11.4 ± 0.32, 12.4 ± 1.76, 10.8 ± 1.22, 16.6 ± 3.84, and 41.2 ± 5.40 RFU, respectively.

**Figure 3:**
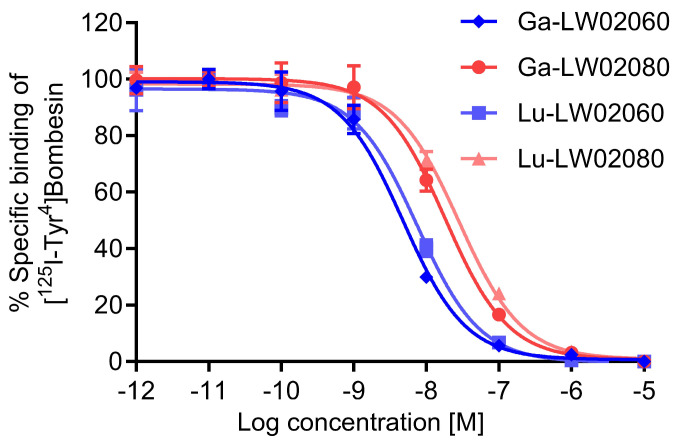
Displacement curves of [^125^I-Tyr^4^]Bombesin by Ga-LW02060, Ga-LW02080, Lu-LW02060, and Lu-LW02080 generated using GRPR-expressing PC-3 cells. Error bars indicate standard deviation (n = 3).

The binding affinities of Ga-LW02060, Ga-LW02080, Lu-LW02060, and Lu-LW02080 were determined by in vitro competition binding assays using GRPR-expressing PC-3 cells and [^125^I-Tyr^4^ ]Bombesin as the radioligand. The binding of [^125^I-Tyr^4^]Bombesin to PC-3 cells was inhibited by all tested ligands in a dose-dependent manner (Figure 3). The calculated K_i_ values for Ga-LW02060, Ga-LW02080, Lu-LW02060, and Lu-LW02080 were 5.57 ± 2.47, 21.7 ± 6.69, 8.00 ± 2.61, and 32.1 ± 8.14 nM, respectively (n = 3).

The hydrophilicity of [^68^Ga]Ga-LW02060, [^68^Ga]Ga-LW02080, [^177^Lu]Lu-LW02060, and [^177^Lu]Lu-LW02080 were determined via the shake flask method, and the calculated logD_7.4_ values were −2.57 ± 0.04, −2.60 ± 0.03, −2.45 ± 0.03, and −2.33 ± 0.26, respectively (n = 3).

### 2.3. PET Imaging and Ex Vivo Biodistribution

**Figure 4:**
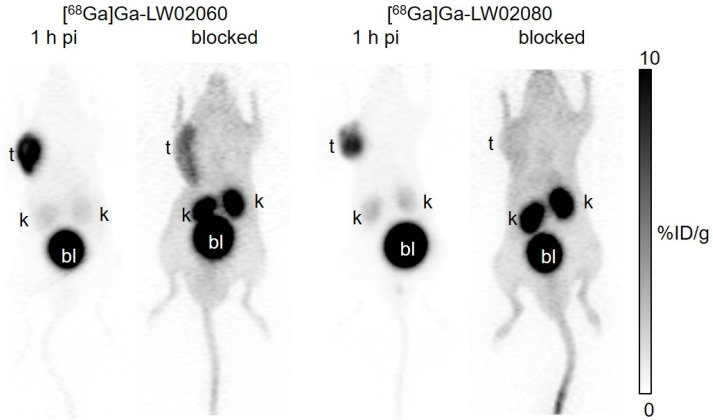
Representative PET images of [^68^Ga]Ga-LW02060 and [^68^Ga]Ga-LW02080 acquired at 1 h pi in PC-3 tumor-bearing mice. The blocked mouse of [^68^Ga]Ga-LW02060 was co-injected with 100 μg of [D-Phe^6^,Leu-NHEt^13^,des-Met^14^]Bombesin(6–14), and the blocked mouse of [^68^Ga]Ga-LW02080 was co-injected with 100 μg of nonradioactive Ga-LW02080. t: tumor; k: kidney; bl: urinary bladder.

Both [^68^Ga]Ga-LW02060 and [^68^Ga]Ga-LW02080 were excreted mainly via the renal pathway and enabled clear visualization of the PC-3 tumor xenografts in PET images at 1 h pi as shown in Figure 4. Except tumor xenografts, kidneys, and the urinary bladder, the uptake of both tracers in other organs/tissues were minimal. [^68^Ga]Ga-LW02060 showed a better tumor uptake, and both tracers had excellent tumor-to-background imaging contrast. Co-injection with 100 μg of [D-Phe^6^,Leu-NHEt^13^,des-Met^14^]Bombesin(6–14) or nonradioactive Ga-LW02080 significantly decreased the uptake of [^68^Ga]Ga-LW02060 and [^68^Ga]Ga-LW02080, respectively, in PC-3 tumor xenografts. The kidney uptake in both blocked mice were markedly increased.

**Figure 5:**
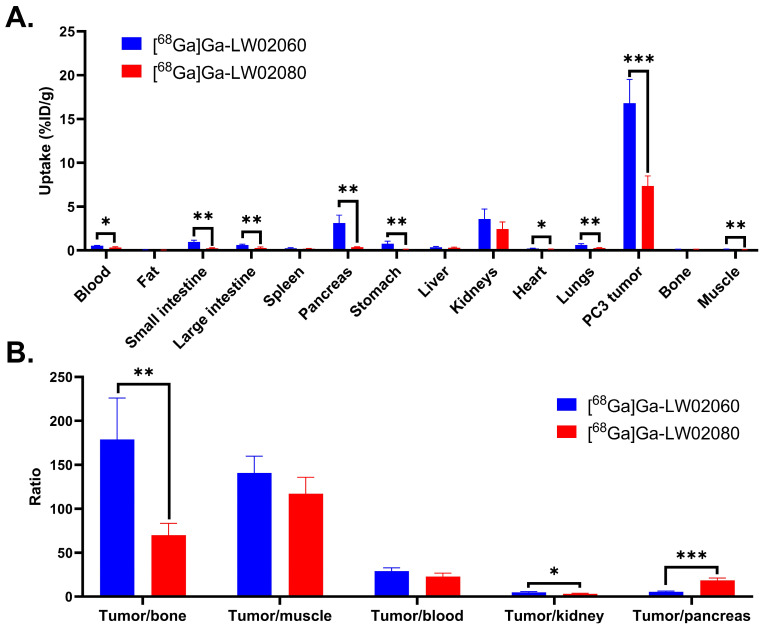
(**A**) Uptake of [^68^Ga]Ga-LW02060 and [^68^Ga]Ga-LW02080 in PC-3 tumor xenografts and major organs/tissues of NRG mice (n = 4) at 1 h pi. Error bars indicate standard deviation. (**B**) Tumor-to-organ uptake ratios of [^68^Ga]Ga-LW02060 and [^68^Ga]Ga-LW02080 obtained from PC-3 tumor-bearing mice (n = 4) at 1 h pi. Error bars indicate standard deviation. * *p* < 0.05; ** *p* < 0.01; *** *p* < 0.001.

**Figure 6:**
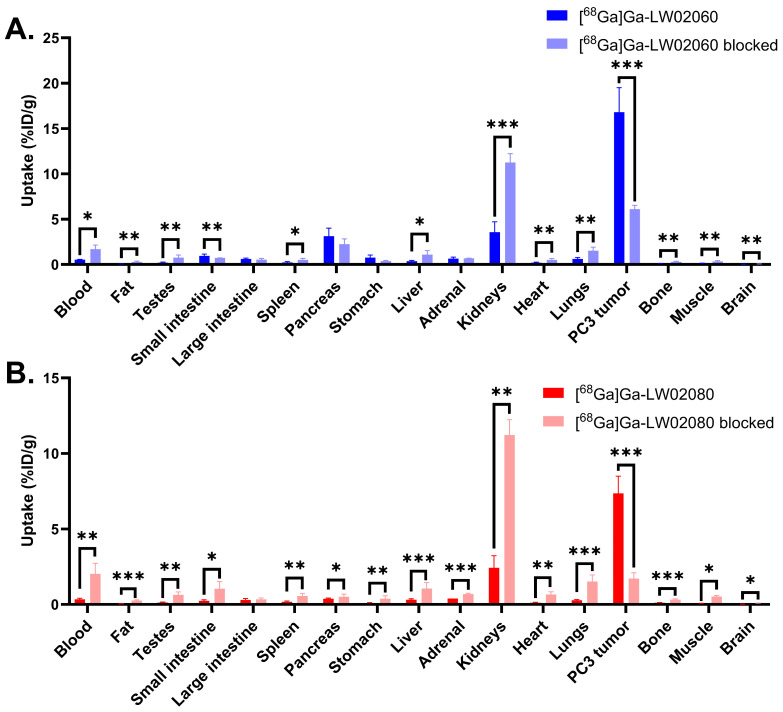
(**A**) Comparison of [^68^Ga]Ga-LW02060 with/without co-injection of [D-Phe^6^,Leu-NHEt^13^,des-Met^14^]Bombesin(6–14) on the uptake in PC-3 tumor xenografts and major organs/tissues in mice (n = 4) at 1 h pi. (**B**) Comparison of [^68^Ga]Ga-LW02080 with/without co-injection of its nonradioactive standard on the uptake in PC-3 tumor xenografts and major organs/tissues in mice (n = 4) at 1 h pi. * *p* < 0.05, ** *p* < 0.01, *** *p* < 0.001.

The ex vivo biodistribution studies for both [^68^Ga]Ga-LW02060 and [^68^Ga]Ga-LW02080 were conducted at 1 h pi in PC-3 tumor-bearing mice, and the results were consistent with the observations from the PET images (Figure 5 and Figure 6 and Table S4). Compared to [^68^Ga]Ga-LW02080, [^68^Ga]Ga-LW02060 had a higher tumor uptake (7.36 ± 1.13 vs. 16.8 ± 2.70 %ID/g, *p* < 0.001), leading to better tumor-to-bone and tumor-to-kidney uptake ratios (69.8 ± 13.7 vs. 179 ± 46.9 and 3.18 ± 0.75 vs. 4.86 ± 0.76, respectively). The pancreas uptake of [^68^Ga]Ga-LW02060 was 3.12 ± 0.89 %ID/g, significantly higher than that of [^68^Ga]Ga-LW02080 (0.38 ± 0.04 %ID/g, *p* < 0.05), resulting in a lower tumor-to-pancreas uptake ratio for [^68^Ga]Ga-LW02060 (5.52 ± 0.78 vs. 18.4 ± 2.86, *p* < 0.001). Furthermore, [^68^Ga]Ga-LW02060 also showed significantly higher uptake in some normal organs than [^68^Ga]Ga-LW02080 such as in small intestine, large intestine, and stomach. Consistent with the observations from PET images, moderate kidney uptake was obtained for [^68^Ga]Ga-LW02060 (3.58 ± 1.14 %ID/g) and [^68^Ga]Ga-LW02080 (2.42 ± 0.81 %ID/g). Except tumor xenografts and kidneys, the uptake values of [^68^Ga]Ga-LW02060 and [^68^Ga]Ga-LW02080 for all other collected organs/tissues were lower than 1 %ID/g.

Co-injection with 100 μg of [D-Phe^6^,Leu-NHEt^13^,des-Met^14^]Bombesin(6–14) significantly reduced the average uptake of [^68^Ga]Ga-LW02060 in PC-3 tumor xenografts by 64% (from 16.8 ± 2.70 %ID/g to 6.11 ± 0.42 %ID/g, *p* < 0.001) (Figure 6A and Table S4). Co-injection with 100 μg of nonradioactive Ga-LW02080 reduced the average tumor uptake of [^68^Ga]Ga-LW02080 by 77% (from 7.36 ± 1.13 %ID/g to 1.71 ± 0.39 %ID/g, *p* < 0.001) (Figure 6B and Table S4). Co-injection with a blocking agent also significantly increased the uptake of both tracers in most of the collected normal organs/tissues, especially in kidneys (from 3.58 ± 1.14 %ID/g to 11.3 ± 0.97 %ID/g for [^68^Ga]Ga-LW02060, *p* < 0.001; from 2.42 ± 0.81 %ID/g to 11.2 ± 1.02 %ID/g for [^68^Ga]Ga-LW02080, *p* < 0.01,) (Figure 6 and Table S4).

### 2.4. In Vivo Stability

**Figure 7:**
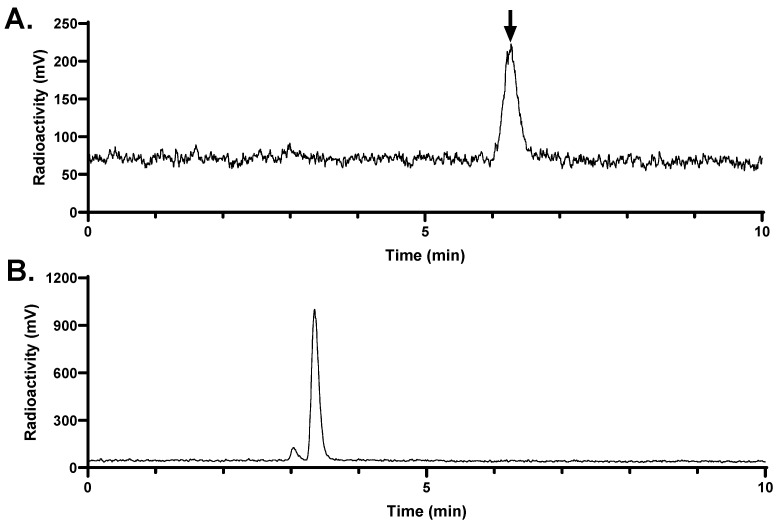
Representative radio-HPLC chromatograms from analysis of intact fraction of [^68^Ga]Ga-LW02060 in mouse (**A**) plasma and (**B**) urine samples collected at 15 min pi. The black arrow points to the peak of intact [^68^Ga]Ga-LW02060.

**Figure 8:**
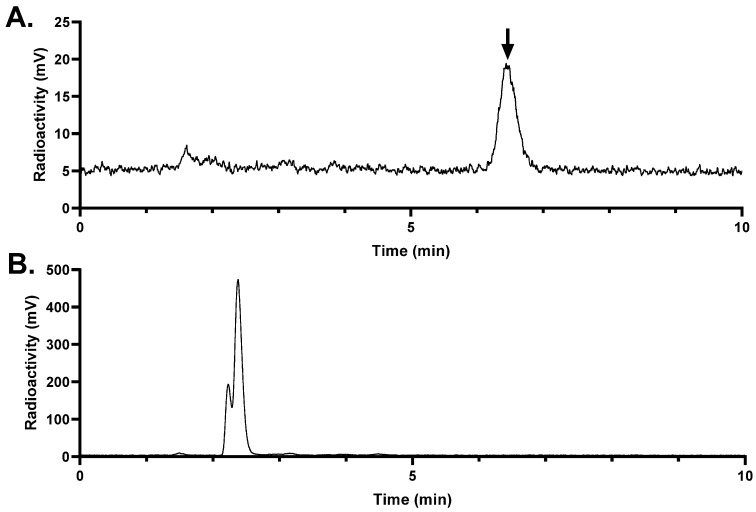
Representative radio-HPLC chromatograms from analysis of intact fraction of [^68^Ga]Ga-LW02080 in mouse (**A**) plasma and (**B**) urine samples collected at 15 min pi. The black arrow points to the peak of intact [^68^Ga]Ga-LW02080.

The in vivo stability of [^68^Ga]Ga-LW02060 and [^68^Ga]Ga-LW02080 was evaluated in NRG mice (n = 3) (Figure 7 and Figure 8). There was >99% of [^68^Ga]Ga-LW02060 remaining intact in plasma at 15 min pi, which was higher than that of [^68^Ga]Ga-LW02080, with 87.4 ± 5.34% remaining intact (*p* < 0.01). No intact tracer was detected in urine samples collected at 15 min pi for either [^68^Ga]Ga-LW02060 or [^68^Ga]Ga-LW02080.

### 2.5. SPECT Imaging and Ex Vivo Biodistribution

**Figure 9:**
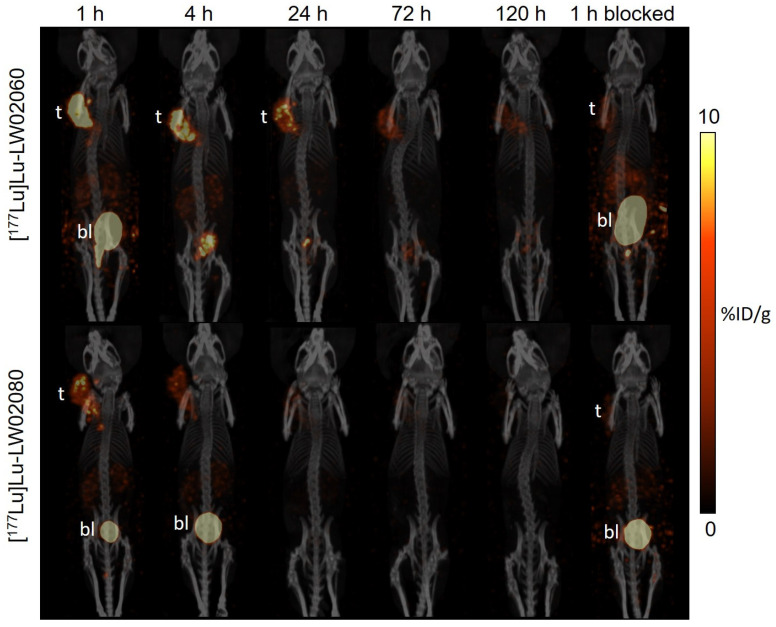
Longitudinal SPECT/CT images of [^177^Lu]Lu-LW02060 (top) and [^177^Lu]Lu-LW02080 (bottom) in PC-3 tumor-bearing NRG mice. Acquisition time points were 1, 4, 24, 72, and 120 h pi. The blocked mice were co-injected with 100 μg of [D-Phe^6^,Leu-NHEt^13^,des-Met^14^]Bombesin(6–14). t: tumor; bl: urinary bladder.

The longitudinal SPECT/CT images of [^177^Lu]Lu-LW02060 and [^177^Lu]Lu-LW02080 were acquired at 1, 4, 24, 72, and 120 h pi (Figure 9). [^177^Lu]Lu-LW02060 enabled clear visualization of the PC-3 tumor xenograft in the SPECT images up to 24 h pi, while [^177^Lu]Lu-LW02080 can be used to clearly visualize the PC-3 tumor xenograft only at 1 h pi. Both [^177^Lu]Lu-LW02060 and [^177^Lu]Lu-LW02080 were excreted mainly via the renal pathway, which resulted in moderate kidney uptake and extremely high accumulation in the urinary bladders and urethras. Co-injection with 100 μg of [D-Phe^6^,Leu-NHEt^13^,des-Met^14^]Bombesin(6–14) significantly decreased the uptake of [^177^Lu]Lu-LW02060 and [^177^Lu]Lu-LW02080 in PC-3 tumor xenografts at 1 h pi.

**Figure 10:**
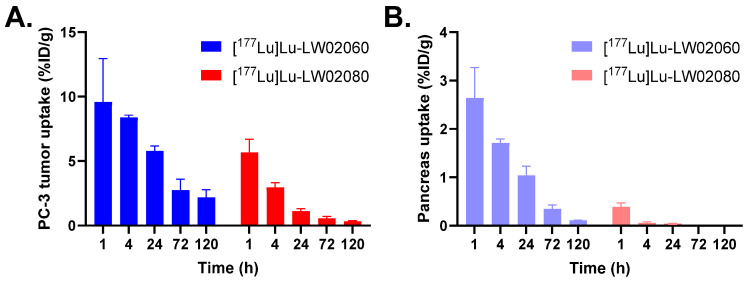
Uptake of [^177^Lu]Lu-LW02060 and [^177^Lu]Lu-LW02080 in (**A**) PC-3 tumor xenografts and (**B**) pancreas at 1, 4, 24, 72, and 120 h pi. Error bars indicate standard deviation (n = 5).

The ex vivo biodistribution results of [^177^Lu]Lu-LW02060 and [^177^Lu]Lu-LW02080 were consistent with the observations from the SPECT images (Figure 9 and Figure 10 and Tables S5 and S6). The tumor uptake values of [^177^Lu]Lu-LW02060 were 9.59 ± 3.37, 8.38 ± 0.19, and 5.78 ± 0.40 %ID/g at 1, 4, and 24 h pi, respectively, and then dropped to <3 %ID/g after 72 h pi. By contrast, the tumor uptake of [^177^Lu]Lu-LW02080 was only 5.67 ± 1.02 %ID/g at 1 h pi and decreased quickly to 2.96 ± 0.37 %ID/g at 4 h pi and <1 %ID/g after 72 h pi (Figure 10A). The pancreas uptake of [^177^Lu]Lu-LW02060 were 2.64 ± 0.63, 1.71 ± 0.08, and 1.04 ± 0.19 %ID/g at 1, 4, and 24 h pi, respectively, while [^177^Lu]Lu-LW02080 showed markedly lower pancreas uptake (<0.5 %ID/g for all time points) (Figure 10B). The kidney uptake values of [^177^Lu]Lu-LW02060 were 3.62 ± 0.86, 2.85 ± 0.29, and 1.06 ± 0.22 %ID/g at 1, 4, and 24 h pi, respectively, and slightly lower kidney uptake values were obtained from [^177^Lu]Lu-LW02080 (2.65 ± 0.48, 1.88 ± 0.35, and 0.89 ± 0.28 %ID/g at 1, 4, and 24 h pi, respectively). Uptake values of [^177^Lu]Lu-LW02060 and [^177^Lu]Lu-LW02080 for all other collected organs/tissues were <1 %ID/g at all time points (Tables S5 and S6).

Co-injection with 100 µg of [D-Phe^6^,Leu-NHEt^13^,des-Met^14^]Bombesin(6–14) reduced the tumor uptake of [^177^Lu]Lu-LW02060 and [^177^Lu]Lu-LW02080 at 1 h pi by 66% and 64%, respectively. For [^177^Lu]Lu-LW02060, significant uptake reductions were also observed for pancreas, stomach, and small intestine by 81%, 72%, and 66%, respectively, with co-injection of the blocking agent. By contract, for [^177^Lu]Lu-LW02080, no significant uptake change was observed for the pancreas (*p* > 0.05), but a slightly higher uptake was found in stomach and small intestine with co-injection of the blocking agent. No significant difference in kidney uptake of [^177^Lu]Lu-LW02060 was observed without/with co-injection of [D-Phe^6^,Leu-NHEt^13^,des-Met^14^]Bombesin(6–14), while a significant increase in the kidney uptake of [^177^Lu]Lu-LW02080 (from 2.65 ± 0.48 %ID/g to 4.17 ± 0.95 %ID/g, *p* < 0.05) was observed.

### 2.6. Radiation Dosimetry

**Figure 11:**
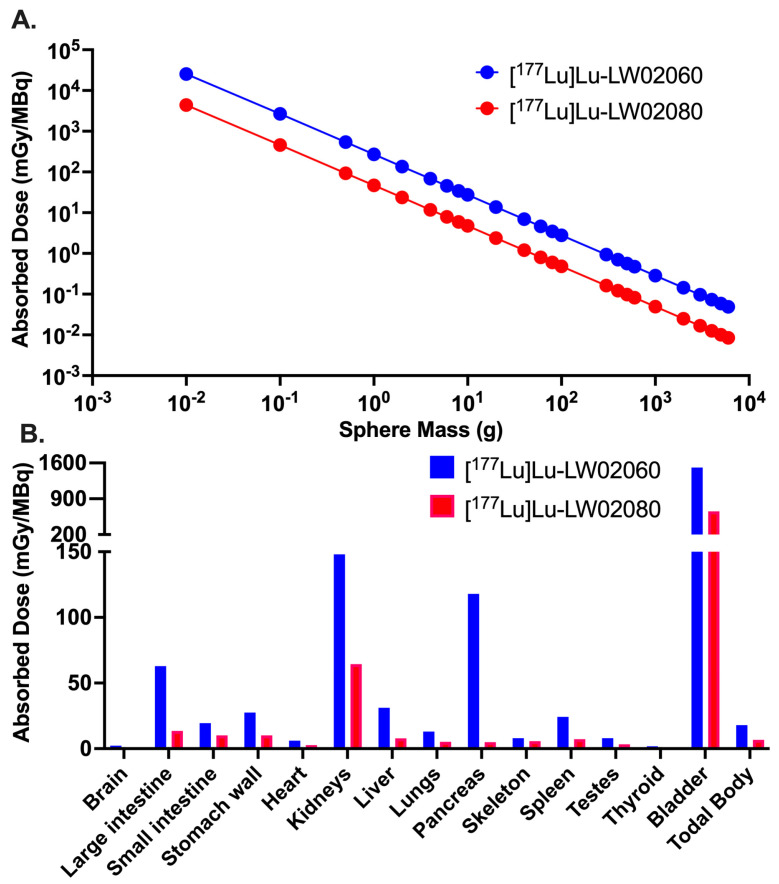
(**A**) Radiation absorbed doses (mGy/MBq) of [^177^Lu]Lu-LW02060 and [^177^Lu]Lu-LW02080 to PC-3 tumor xenografts, obtained with various tumor masses but assuming the same tumor uptake (%ID) and residence time for [^177^Lu]Lu-LW02060 and [^177^Lu]Lu-LW02080. (**B**) Comparison of radiation absorbed doses with the tumor sink effect correction for selected mouse organs/tissues per unit of injected activity (mGy/MBq) from [^177^Lu]Lu-LW02060 and [^177^Lu]Lu-LW02080.

The calculated radiation doses absorbed by selected mouse organs/tissues from [^177^Lu]Lu-LW02060 and [^177^Lu]Lu-LW02080 are provided in Figure 11 and Tables S7 and S8. A higher radiation absorbed dose of [^177^Lu]Lu-LW02060 was delivered to the PC-3 tumor xenografts (Unit Density Sphere Model) compared with that of [^177^Lu]Lu-LW02080 (Figure 11A). The absorbed dose of [^177^Lu]Lu-LW02060 in a 1-g PC-3 tumor xenograft was 272 mGy/MBq, which was 5.8-times the tumor absorbed dose of [^177^Lu]Lu-LW02080 (47.0 mGy/MBq) (Figure 11A and Table S7). The urinary bladder received the highest absorbed dose for both [^177^Lu]Lu-LW02060 and [^177^Lu]Lu-LW02080 among all selected organs/tissues with 1510 and 655 mGy/MBq, respectively, followed by the kidneys, with 148 and 64.4 mGy/MBq, respectively (Figure 11B and Table S7). Compared with [^177^Lu]Lu-LW02060, an overall less absorbed dose was observed for [^177^Lu]Lu-LW02080 in all selected organs/tissues. The absorbed dose of [^177^Lu]Lu-LW02060 in the pancreas was 118 mGy/MBq, which was around 23-times the pancreas absorbed dose of [^177^Lu]Lu-LW02080 (5.03 mGy/MBq).

The estimated radiation absorbed doses with the tumor sink effect correction for both [^177^Lu]Lu-LW02060 and [^177^Lu]Lu-LW02080 in an average adult male are provided in Table 1. The highest estimated absorbed dose was observed in the urinary bladder for both ^177^Lu-labeled ligands, with 1.50 × 10^−1^ and 6.51 × 10^−2^ mGy/MBq, respectively. The estimated absorbed dose of [^177^Lu]Lu-LW02060 in the pancreas (5.08 × 10^−2^ mGy/MBq) was around 30 times of the pancreas absorbed dose of [^177^Lu]Lu-LW02080 (1.69 × 10^−3^ mGy/MBq). The estimated absorbed doses of [^177^Lu]Lu-LW02060 were consistently higher than those of [^177^Lu]Lu-LW02080 across other selected organs/tissues, ranging from 1.9-fold to 5.8-fold. The effective whole-body dose per injected radioactivity of [^177^Lu]Lu-LW02060 and [^177^Lu]Lu-LW02080 to an adult human male were 1.16 × 10^−2^ and 4.15 × 10^−3^ mSv/MBq, respectively.

## 3. Discussion

Based on our previously reported LW02056 and ProBOMB5, we designed two novel DOTA-conjugated GRPR-targeted ligands, LW02060 and LW02080, by replacing Pro^14^ in LW02056 and ProBOMB5, respectively, with diF-Pro^14^ (Figure 1) [24]. The data from intracellular calcium efflux assay confirm the agonist characteristics of LW02060 and its Ga/Lu-complexed analogs, and the antagonist characteristics of LW02080 and its Ga/Lu-complexed analogs (Figure 2). The agonist/antagonist characteristics of both diF-Pro^14^ derivatives are aligned with their Pro^14^ analogs (LW02056 and ProBOMB5). This validates our hypothesis that diF-Pro^14^ substitution on previously reported LW02056 and ProBOMB5 would retain their GRPR agonist/antagonist characteristics

The binding affinities of the Ga/Lu-complexed LW02060 analogs were better than those of LW02080, respectively (Figure 3). Compared to its Pro^14^ analog Ga-LW02056, Ga-LW02060 showed a significantly higher binding affinity to GRPR (K_i_ = 14.7 ± 4.81 vs. 5.57 ± 2.47 nM, *p* < 0.05) [24]. In contrast, the binding affinities of the GRPR antagonists, Ga-LW02080 and Lu-LW02080, were found to be lower than those of Ga-ProBOMB5 and Lu-ProBOMB5 (K_i_ = 21.7 ± 6.69 and 32.1 ± 8.14 nM vs. 12.2 ± 1.89 and 13.6 ± 0.25 nM, respectively) [24]. This observation suggests that the diF-Pro^14^ substitution enhances the binding affinity of GRPR agonists (Ga-LW02060) while reducing the binding affinity of GRPR antagonists (Ga-LW02080 and Lu-LW02080). Previously, we also observed that NMe-Gly^11^ substitution was tolerable for antagonists but decreased the binding affinity of agonists [20,23]. Similarly, NMe-His^12^ substitution was found to improve binding affinity for only agonists but to decrease the binding affinity for antagonists [22]. Our accumulated data suggest that agonists and antagonists bind to GRPR in different configurations.

The hydrophilic nature of [^68^Ga]Ga-LW02060, [^68^Ga]Ga-LW02080, [^177^Lu]Lu-LW02060, and [^177^Lu]Lu-LW02080 were confirmed via the logD_7.4_ measurements with logD_7.4_ values all ≤−2.33. Compared with their Pro^14^ analogs ([^68^Ga]Ga-LW02056 and [^68^Ga]Ga-ProBOMB5), both [^68^Ga]Ga-LW02060 and [^68^Ga]Ga-LW02080 had significantly higher logD_7.4_ values (−2.93 ± 0.08 and −2.74 ± 0.04 vs. −2.57 ± 0.04 and −2.60 ± 0.03, respectively; *p* < 0.01). This suggests that replacing Pro^14^ with diF-Pro^14^ slightly increases the lipophilicity of two tracers.

Both [^68^Ga]Ga-LW02060 and [^68^Ga]Ga-LW02080 enabled clear visualization of the PC-3 tumor xenografts in PET images at 1 h pi (Figure 4). The extremely high accumulation of both tracers in the urinary bladders seen in PET images indicates that their main excretion was via the renal pathway, likely resulting from the highly hydrophilic nature of both tracers. The ex vivo biodistribution data of [^68^Ga]Ga-LW02060 and [^68^Ga]Ga-LW02080 were consistent with the observations from their PET images (Figure 4, Figure 5 and Figure 6 and Table S4). Possibly owing to its higher binding affinity, [^68^Ga]Ga-LW02060 exhibited 2.3-fold uptake in PC-3 tumor xenografts when compared to that of [^68^Ga]Ga-LW02080 (16.8 ± 2.70 vs. 7.36 ± 1.13 %ID/g), and 1.9-fold uptake when compared to the previously reported [^68^Ga]Ga-LW02056 (16.8 ± 2.70 vs. 8.93 ± 1.96 %ID/g) [24]. On the other hand, compared with [^68^Ga]Ga-ProBOMB5, the diF-Pro^14^-derived [^68^Ga]Ga-LW02080 showed a significantly lower tumor uptake (7.36 ± 1.13 vs. 12.4 ± 1.35 %ID/g, *p* < 0.005), likely due to the inferior GRPR binding affinity of Ga-LW02080 [24].

To avoid the potential toxicity caused by injecting an excessive amount of a GRPR agonist, we conducted the blocking study of the agonist tracer, [^68^Ga]Ga-LW02060, by co-injection with 100 μg of a GRPR antagonist, [D-Phe^6^,Leu-NHEt^13^,des-Met^14^]Bombesin(6–14) (Figure 4 and Figure 6, and Table S4). For the antagonist tracer, [^68^Ga]Ga-LW02080, the blocking study was conducted with the co-injection of its nonradioactive standard, Ga-LW02080 (100 μg) (Figure 4 and Figure 6 and Table S4). With a significantly reduced tumor uptake in the blocked mice, both blocking studies confirmed the specific uptake of [^68^Ga]Ga-LW02060 and [^68^Ga]Ga-LW02080 in the GRPR-expressing PC-3 tumor xenografts. The increased background uptake especially in the kidneys of blocked mice mainly resulted from the competitive binding of blocking agents to the GRPR in PC-3 tumor xenografts, leading to more unbound [^68^Ga]Ga-LW02060 and [^68^Ga]Ga-LW02080 in the blood pool and to be excreted through the renal pathway.

The in vivo stability of [^68^Ga]Ga-LW02060 and [^68^Ga]Ga-LW02080 was assessed at 15 min pi (Figure 7 and Figure 8). More than 99% of [^68^Ga]Ga-LW02060 remained intact in mouse plasma, which was significantly higher than that of [^68^Ga]Ga-LW02080 (87.4 ± 5.34%) and our previously reported GRPR-targeted tracers (12.7–92.9%) [20,21,22,23,24]. The excellent in vivo stability of [^68^Ga]Ga-LW02060 likely contributes to its high tumor uptake (16.8 ± 2.70 at 1 h pi). Same as most of the previously reported GRPR-targeted radioligands, no intact tracer was observed in mouse urine samples, likely due to degradation by NEP, which is expressed abundantly in the kidneys [18,19,20,21,22,23,24].

Subsequently, we labeled LW02060 and LW02080 with Lu-177 and evaluated the therapeutic potential of their ^177^Lu-labeled analogs via SPECT/CT imaging, ex vivo biodistribution studies, and dosimetry calculations. The longitudinal SPECT/CT images showed that compared with [^177^Lu]Lu-LW02060, [^177^Lu]Lu-LW02080 had a lower tumor uptake and was cleared faster from the PC-3 tumor xenograft (Figure 9). This is likely due to the combination of its inferior GRPR binding affinity and being an antagonist (lack of internalization upon binding to GRPR) [1,29,30]. The observations from the SPECT/CT images were confirmed by data from ex vivo biodistribution studies (Figure 10 and Tables S5 and S6). The tumor uptake of [^177^Lu]Lu-LW02060 and [^177^Lu]Lu-LW02080 at 1 h pi was lower than that of their ^68^Ga-labeled analogs, [^68^Ga]Ga-LW02060 and [^68^Ga]Ga-LW02080 (9.59 ± 3.37 and 5.67 ± 1.02 %ID/g vs. 16.8 ± 2.70 and 7.36 ± 1.13 %ID/g, respectively). The inferior tumor uptake for the ^177^Lu-labeled analogs could be due to their slightly inferior GRPR binding affinity, possibly resulting from variation in the charge distribution of different DOTA-metal complexes. It has been previously reported by Fani et al. that complexation with a different metal could have a high impact on the binding affinity of a somatostatin receptor 2 (Sstr2)-targeted ligand, DOTA-JR11 [31]. While the IC_50_ values of Y-DOTA-JR11 and Lu-DOTA-JR11 were 0.47 and 0.73 nM, respectively, the IC_50_ value of Ga-DOTA-JR11 was only 29 nM. Surprisingly, with a different chelator, Ga-NODAGA-JR11 was much more potent and had an IC_50_ value at 1.2 nM, close to that of Y-DOTA-JR11 and Lu-DOTA-JR11. Therefore, we are currently investigating the effect of different chelators on the binding affinity of Ga/Lu-complexed GRPR-targeted ligands.

Owing to their dominantly renal excretion, the highest absorbed dose for both [^177^Lu]Lu-LW02060 and [^177^Lu]Lu-LW02080 was received by the urinary bladder in the mouse model. The calculated absorbed dose of [^177^Lu]Lu-LW02060 in a 1-g PC-3 tumor xenograft was 272 mGy/MBq, which was 5.8-times that of [^177^Lu]Lu-LW02080 (47.0 mGy/MBq) and 4.7-time the tumor absorbed dose of our previously reported [^177^Lu]Lu-ProBOMB5 (57.3 mGy/MBq) [24]. However, the absorbed dose of the clinical validated GRPR antagonist [^177^Lu]Lu-RM2 in the same tumor model was 429 mGy/MBq, which is 1.6-times that of [^177^Lu]Lu-LW02060. This is due to the longer tumor retention of [^177^Lu]Lu-RM2, likely resulting from its higher GRPR binding affinity (K_i_ = 1.19 ± 0.16 nM) [24]. The radiation absorbed doses delivered to the pancreas by [^177^Lu]Lu-LW02060 and [^177^Lu]Lu-LW02080 (118 and 5.03 mGy/MBq) were markedly lower than that of [^177^Lu]Lu-RM2 (316 mGy/MBq) [24]. The estimated absorbed doses of [^177^Lu]Lu-LW02060 and [^177^Lu]Lu-LW02080 in the human pancreas were markedly lower than those of [^177^Lu]Lu-RM2 (5.08 × 10^−2^ and 1.69 × 10^−3^ mGy/MBq, respectively, vs. 1.16 × 10^−1^ mGy/MBq) [24]. Because the pancreas is the main dose-limiting organ of GRPR-targeted radiopharmaceuticals [9,10], our data suggested that [^177^Lu]Lu-LW02060 and [^177^Lu]Lu-LW02080 have higher maximum tolerated doses compared with the clinically validated [^177^Lu]Lu-RM2 owing to their relatively lower pancreas accumulation. Additionally, the overall lower absorbed doses of [^177^Lu]Lu-LW02060 and [^177^Lu]Lu-LW02080 in most selected organs/tissues compared to those of [^177^Lu]Lu-RM2 indicate less off-target binding of [^177^Lu]Lu-LW02060 and [^177^Lu]Lu-LW02080 in both the mouse model and adult human.

Though compared to [^177^Lu]Lu-LW02080, [^177^Lu]Lu-LW02060 has a higher tumor uptake and a longer tumor retention, it is still not ideal for therapeutic application due to its insufficient radiation absorbed dose deposited in tumors. Further optimizations are needed to enhance tumor uptake and extend tumor retention, thereby achieving a potentially better therapeutic efficacy.

## 4. Materials and Methods

### 4.1. General Methods

All chemicals and solvents used in this study were obtained from commercial suppliers and utilized without additional purification. GRPR-targeting peptides were synthesized using solid phase approach on an AAPPTec (Louisville, KY, USA) Endeavor 90 peptide synthesizer. Purification and quality control of synthesized peptides were performed using Agilent (Santa Clara, CA, USA) HPLC systems equipped with a model 1200 quaternary pump, a model 1200 UV absorbance detector (220 nm), and a Bioscan (Washington, DC, USA) NaI scintillation detector. The Agilent HPLC systems were operated using the Agilent ChemStation software. The HPLC columns utilized in this study included a semi-preparative column (Luna C18, 5 µm, 250 × 10 mm) and an analytical column (Luna C18, 5 µm, 250 × 4.6 mm), both acquired from Phenomenex (Torrance, CA, USA). The collected HPLC eluates were lyophilized using a Labconco (Kansas City, MO, USA) FreeZone 4.5 Plus freeze-drier. MS spectra were obtained using an AB SCIEX (Framingham, MA, USA) 4000 QTRAP mass spectrometer system equipped with an ESI ion source. C18 Sep-Pak cartridges (1 cm^3^, 50 mg) were purchased from Waters (Milford, MA, USA). ^68^Ga was eluted from an ITM Medical Isotopes GmbH (Munich, Germany) generator and purified according to the previously published procedures using a DGA resin column from Eichrom Technologies LLC (Lisle, IL, USA) [24]. ^177^LuCl_3_ was purchased from Isotopia Molecular Imaging Ltd. (Petah Tikva, Israel) and ITM Medical Isotopes GmbH (Munich, Germany). [^125^I-Tyr^4^]Bombesin was purchased from Revvity (Waltham, MA, USA). Radioactivity of ^68^Ga/^177^Lu-labeled peptides was measured using a Capintec (Ramsey, NJ, USA) CRC^®^-25R/W dose calibrator. The radioactivity of samples collected from biodistribution studies, binding assays, and logD_7.4_ measurements was counted on a Perkin Elmer (Waltham, MA, USA) Wizard2 2480 automatic gamma counter. ^19^F NMR is externally referenced to CFCl_3_ to 0.00 ppm for all NMR samples.

### 4.2. Synthesis of Fmoc-Leu(ψ)diF-Pro-OH

Fmoc-Leu(ψ)diF-Pro-OH (**5**) was synthesized following the reaction steps depicted in Scheme S1. Detailed synthesis procedures and characterizations for Fmoc-Leu(ψ)diF-Pro-OH (**5**) and its intermediates are provided in Supplementary Materials (Figures S1–S16).

### 4.3. Synthesis of DOTA-Conjugated Peptides

Both LW02060 and LW02080 were synthesized on solid phase using the Fmoc peptide chemistry. For the synthesis of LW02060, Rink Amide MBHA resin (0.1 mmol) was deprotected by treating it with 20% piperidine in *N*,*N*-dimethylformamide (DMF) to remove the Fmoc group. After being pre-activated with HATU (5 eq.), HOAt (5 eq.), and *N*,*N*-diisopropylethylamine (DIEA, 15 eq.), Fmoc-protected amino acids (5 eq.) and Fmoc-4-amino-(1-carboxymethyl)piperidine (5 eq.) were sequentially coupled to the resin. Sieber resin (0.1 mmol) was used for the synthesis of LW02080 following the same procedure as LW02060. At the end, DOTA(*t*Bu)_3_ (5 eq.), pre-activated with HATU (5 eq.) and DIEA (25 eq.), was coupled to the resins for both LW02060 and LW02080, respectively.

A mixture of trifluoroacetic acid (TFA, 81.5%), triisopropylsilane (TIS 1.0%), water (5%), 2,2′-(ethylenedioxy)diethanethiol (DODT, 2.5%), thioanisole (5%), and phenol (5%) was used to deprotect and cleave the crude peptides from the resins respectively. After 4 h incubation at room temperature, the cleaved peptides were filtered and precipitated in cold diethyl ether. The crude peptides were collected after centrifugation and purified with HPLC (semi-preparative column; flow rate: 4.5 mL/min). The fractions containing the target peptides were gathered and lyophilized. The HPLC conditions, retention times, isolated yields and MS confirmations of LW02060 and LW02080 are detailed in Table S1 and Figures S17 and S18.

### 4.4. Synthesis of Nonradioactive Ga/Lu-Complexed Standards

The nonradioactive Ga-complexed standards of LW02060 and LW02080 were synthesized by incubating a solution of the DOTA-conjugated precursor with excess GaCl_3_ (5 eq.) in NaOAc buffer (0.1 M, 500 µL, pH 4.5) at 80 °C for 15 min. The nonradioactive Lu-complexed standards of LW02060 and LW02080 were synthesized by incubating a solution of the precursor with excess LuCl_3_ (10 eq.) in NaOAc buffer (0.1 M, 500 µL, pH 4.5) at 90 °C for 30 min. Subsequently, the reaction mixture was purified using HPLC with a semi-preparative column at flow rate of 4.5 mL/min. The HPLC conditions, retention times, isolated yields and MS confirmations of these nonradioactive Ga/Lu-complexed standards are detailed in Table S2 and Figures S19–S22.

### 4.5. Synthesis of ^68^Ga/^177^Lu-Labeled Ligands

The Ga-68 radiolabeling experiments were performed following previously published procedures [24]. Briefly, purified ^68^Ga (158–523 MBq) in 0.5 mL water was added into a 4-mL glass vial containing 0.7 mL of HEPES buffer (2 M, pH 5.0) and 10 μL of precursor solution (1 mM). The radiolabeling reaction was performed by microwave heating for 1 min at 100 °C, followed by purification using HPLC with a semi-preparative column.

The Lu-177 radiolabeling experiments were conducted following literature procedures [24]. ^177^LuCl_3_ (994–2240 MBq) was added into a 4 mL glass vial preloaded with 0.7 mL of NaOAc buffer (0.1 M, pH 4.5) and 10 μL precursor solution (1 mM) and incubated at 95 °C for 15 min with a heating block. ^177^Lu-labeled ligands were purified using HPLC with a semi-preparative column.

The fraction containing the radiolabeled product was collected, diluted with 50 mL of water, and concentrated in a C18 Sep-Pak cartridge, which was pre-washed with ethanol (10 mL) and water (10 mL). The trapped ^68^Ga/^177^Lu-labeled product on the cartridge was eluted off with ethanol (0.4 mL) and diluted with PBS for imaging and biodistribution studies. The HPLC conditions and retention times for the purification and quality control of ^68^Ga/^177^Lu-labeled ligands are provided in Table S3. The representative analytical radio-HPLC chromatograms of the purified ^68^Ga/^177^Lu-labeled ligands are provided in Figures S23–S26.

### 4.6. The LogD_7.4_ Measurement

The logD_7.4_ values of ^68^Ga/^177^Lu-labeled ligands were measured using the previously reported shake flask method [24]. An aliquot of the ^68^Ga/^177^Lu-labeled ligand was added into a 15 mL falcon tube containing a mixture of n-octanol (3 mL) and DPBS (3 mL, pH 7.4). The mixture was mixed by 1 min vortexing, followed by 15 min centrifugation at 3000 rpm. Samples (1 mL each) from the n-octanol and DPBS layers were collected and then measured using a gamma counter (n = 3). The logD_7.4_ value was calculated using the following equation:

$${logD}_{7.4}={log}_{10}[\frac{{counts}_{octanol\;phase}}{{counts}_{buffer\;phase}}].$$

### 4.7. Fluorometric Calcium Release Assay

The agonist/antagonist characteristics of LW02060, Ga-LW02060, Lu-LW02060, LW02080, Ga-LW02080, and Lu-LW02080 were determined following published procedures [24]. The PC-3 cells were obtained from ATCC (via Cedarlane, Burlington, ON, Canada). A 96-well clear bottom black plate was seeded with 5 × 10^4^ PC-3 cells in 100 μL growth media per well 24 h before the assay. The loading buffer (100 μL/well) containing a calcium-sensitive dye (FLIPR Calcium 6 assay kit from Molecular Devices, San Jose, CA, USA) was added into the 96-well plate, followed by 1 h incubation at 37 °C. The plate was then transferred into a FlexStation 3 microplate reader (Molecular Devices, San Jose, CA, USA). LW02060 (50 nM), Ga-LW02060 (50 nM), Lu-LW02060 (50 nM), LW02080 (50 nM), Ga-LW02080 (50 nM), Lu-LW02080 (50 nM), [D-Phe^6^,Leu-NHEt^13^,des-Met^14^]Bombesin(6–14) (50 nM, antagonist control), bombesin (50 nM, agonist control), adenosine triphosphate (ATP, 50 nM, positive control), or DPBS (blank control) was added, and the fluorescent signals were measured for 2 min (λ_Ex_ = 485 nm; λ_Em_ = 525 nm; n = 4). The relative fluorescent units (RFU = max − min) were calculated to determine the agonistic/antagonistic characteristics of LW02060, Ga-LW02060, Lu-LW02060, LW02080, Ga-LW02080, and Lu-LW02080, respectively.

### 4.8. In Vitro Competition Binding Assay

Inhibition constants (K_i_) of GRPR-targeted ligands to GRPR were measured via in vitro competition binding assay using PC-3 cells and [^125^I-Tyr^4^]Bombesin as the radioligand following the previously reported method [24]. PC-3 cells were seeded in 24-well poly-D-lysine plates at 2 × 10^5^ cells/well 48 h before the assay. The growth medium was removed before adding 400 μL of reaction medium (RPMI 1640 containing 2 mg/mL BSA, and 20 mM HEPES). After incubating at 37 °C for 1 h, Ga-LW02060, Ga-LW02080, Lu-LW02060, and Lu-LW02080 in 50 μL of reaction medium with decreasing concentrations (10 μM to 1 pM) and 50 μL of 0.01 nM [^125^I-Tyr^4^]Bombesin were added into the wells then incubated with gentle agitation for 1 h at 37 °C. The cells were carefully washed twice with ice-cold PBS, harvested using trypsinization, and the radioactivity was measured using a Perkin Elmer (Waltham, MA) Wizard2 2480 automatic gamma counter. Data were analyzed using nonlinear regression (one binding site model for competition assay) with GraphPad (San Diego, CA, USA) Prism 10 software (Version 10.1.1).

### 4.9. PET/CT Imaging and Ex Vivo Biodistribution

PET/CT imaging and biodistribution studies were conducted using male NOD.Cg-Rag1^tm1Mom^ Il2rg^tm1Wjl^/SzJ (NRG) mice following previously published procedures [24]. The studies were carried out in compliance with the guidelines established by the Canadian Council on Animal Care and approved by Animal Ethics Committee of the University of British Columbia (protocol number A20-0113, approved on 30 September 2022). The mice were anaesthetized and subcutaneously implanted with 5 × 10^6^ PC-3 cells (100 µL; 1:1 PBS/Matrigel) behind the left shoulder. PET/CT imaging and biodistribution studies were performed once the tumors reached a diameter of 5–8 mm, typically after approximately 4 weeks of growth.

PET/CT imaging experiments were conducted using a Siemens (Knoxville, TN, USA) Inveon micro PET/CT scanner. Around 3–5 MBq of the ^68^Ga-labeled tracer was injected into the mice via lateral caudal tail veins under anaesthesia. The mice were then recovered and roam freely in their cages. A 10 min CT scan was conducted initially for localization, attenuation correction, and reconstructing the PET images at 50 min post-injection, followed by a 10 min static PET imaging acquisition.

For ex vivo biodistribution studies, the mice were injected with the ^68^Ga-labeled tracer (2–4 MBq) via a lateral caudal tail vein. For blocking [^68^Ga]Ga-LW02060, the mice were co-injected with 100 μg of [D-Phe^6^,Leu-NHEt^13^,des-Met^14^]Bombesin(6–14). For blocking [^68^Ga]Ga-LW02080, the mice were co-injected with 100 μg of its nonradioactive standard Ga-LW02080. At 1 h pi, the mice were euthanized by CO_2_ inhalation. Blood and organs/tissues of interest were collected, weighed, and counted using a gamma counter.

### 4.10. SPECT/CT Imaging and Ex Vivo Biodistribution

SPECT/CT imaging was conducted using an MI Labs (Houten, The Netherlands) U-SPECT-II/CT scanner with a custom-made ultra-high sensitivity big mouse collimator (2 mm pinhole size) following published procedure [24]. The experiments were obtained in accordance with the same protocol established in the PET/CT imaging and ex vivo biodistributions section. The mice were used for SPECT/CT imaging and ex vivo biodistributions studies once the tumors reached a diameter of 5–8 mm. PC-3 tumor-bearing mice were sedated (2.5% isoflurane in O_2_) and injected with 31.4–36.9 MBq of the ^177^Lu-labeled ligand through a lateral caudal tail vein. The mouse was imaged at 1, 4, 24, 72, and 120 h pi. At each time point, a 5 min CT scan was performed with 615 μA and 60 kV parameters for localization and attenuation, followed by two 30 min static SPECT scans in list mode with an energy window centered around 208 keV. The U-SPECT II software was used to reconstruct data, and the images were decay corrected to the time of injection with PMOD v 3.402 (PMOD Technologies GmbH, Fallanden, Switzerland).

For ex vivo biodistribution studies, the mice were injected with ~3–5 MBq of the ^177^Lu-labeled ligand (n = 5). At 1, 4, 24, 72, and 120 h post-injection, the mice were anesthetized with 2% isoflurane, euthanized by CO_2_ inhalation, and the tissues/organs of interest were corrected for radioactivity counting. The blocking study was performed at 1 h pi via co-injection of the ^177^Lu-labeled ligand with 100 μg of [D-Phe^6^,Leu-NHEt^13^,des-Met^14^]Bombesin(6–14).

### 4.11. In Vivo Stability Study

For in vivo stability studies, [^68^Ga]Ga-LW02060 or [^68^Ga]Ga -LW02080 (5.23–12.9 MBq) was injected into healthy male NRG mice (n = 3) tough a lateral caudal tail vein. At 15 min pi, mice were anaesthetized and euthanized, followed by collection of the urine and blood samples. The plasma was extracted from whole blood by adding an equal volume of CH_3_CN, followed by vortexing, centrifugation, and collection of the supernatant. The plasma and urine samples were analyzed via radio-HPLC.

### 4.12. Dosimetry Analysis

The uptake values (%ID/g) obtained from ex vivo biodistribution studies (n = 5) were decayed corrected to the appropriate time point and fitted to mono- or bi-exponential equations using SciPy library integrated into an in-house Python script (Python Software Foundation v.3.10.12) [32]. The best fit was selected based on maximizing the coefficient of determination (R^2^) and minimizing the residuals. Time–activity curves calculated from the parameters obtained from the best fit for each organ were then integrated and normalized to injected activity to acquire time-integrated activity coefficients (TIACs) per unit gram, and subsequently multiplied by the mass of model tissue (30-g mouse phantom) [33]. The TIACs were corrected for the tumor sink effect following formula adopted in the report by Cicone et al. [34], as shown below:

$${TIAC}_{m,corrected}\left(organ\right)={TIAC}_{m}\left(organ\right)+\left\lceil {TIAC}_{m}\left(tumor\right)\times \frac{{TIAC}_{m}\left(organ\right)}{{TIAC}_{m}\left(WB\right){-TIAC}_{m}\left(tumor\right)}\right\rceil .$$

The TIAC values were input into OLINDA (Hermes Medical Solutions, v2.2.3) software [35], which has pre-calculated dose factors for mouse models. Mouse biodistribution data were extrapolated to humans using the method published by Kirschner et al. using the following equation [36]:

$${TIAC}_{H}\left(organ\right)={TIAC}_{M}\left(organ\right)\times \left\lceil \frac{{m(organ)}_{H}/{WB}_{H}}{{m(organ)}_{M}/{WB}_{M}}\right\rceil .$$

m(organ)_H_ and m(organ)_M_ are masses of human and mouse organs, respectively, and *WB* stands for total-body mass. Human TIACs calculated with the above equation were input into OLINDA, and dosimetry results were assessed for ICRP 89 Adult Male Model [37]. The %ID/g value for the blood was assumed to be that for the heart contents of the phantom. Lastly, the TIAC for the tumor was also calculated on the basis of the biodistribution data, and the values were input into the sphere model available in OLINDA [38].

### 4.13. Statistical Analysis

Statistical analyses were performed by using Student’s *t*-test with the Microsoft (Redmond, WA, USA) Excel software. The unpaired two-tailed test was used to compare biodistribution data, binding affinities, and logD_7.4_ values of two radioligands. The unpaired one-tailed test was used to compare the biodistribution data of unblocked mice with those of blocked mice. A statistically significant difference was determined when the adjusted *p* value was <0.05.

## 5. Conclusions

We synthesized two novel GRPR-targeted ligands, LW02060 and LW02080, by replacing Pro^14^ in previously reported LW02056 (an agonist) and ProBOMB5 (an antagonist), respectively, with diF-Pro^14^. Our study reveals that the diF-Pro^14^ substitution retains their agonist/antagonist characteristics. The diF-Pro^14^ substitution on the agonist sequence further improves binding affinity and in vivo stability. By contrast, the diF-Pro^14^ substitution significantly reduces the binding affinity of the antagonist ligand which has a reduced peptide bond (CH_2_-N) between Leu^13^ and AA^14^. Consistent with the low pancreas uptake of their previously reported Pro^14^ analogs, both [^68^Ga]Ga-LW02060 and [^68^Ga]Ga-LW02080 showed low pancreas uptake compared to the clinically evaluated GRPR-targeted tracers. With high in vivo stability, great tumor uptake, and excellent tumor-to-background imaging contrast, [^68^Ga]Ga-LW02060 is a promising tracer for clinical translation to detect GRPR-expressing cancer.

In addition, we successfully labeled both GRPR-targeted ligands with Lu-177 and evaluated the therapeutic potential of the resulting [^177^Lu]Lu-LW02060 and [^177^Lu]Lu-LW02080. However, moderate tumor uptake and rapid clearance from PC-3 tumor xenografts were observed for both [^177^Lu]Lu-LW02060 and [^177^Lu]Lu-LW02080, resulting in low radiation absorbed doses delivered to tumors. Thus, further optimizations are still needed for both ligands to enhance tumor uptake and extend tumor retention for potential therapeutic applications.

## 6. Patents

The compounds disclosed in this report are covered by a recent patent application (PCT International Application Serial No. PCT/CA2024/051215; filing date: 13 September 2024). Lei Wang, Chao-Cheng Chen, François Bénard, and Kuo-Shyan Lin are listed as inventors of this filed patent.

## Figures and Tables

**Table 1:** Estimated radiation absorbed doses (mGy/MBq) in adult human males for [^177^Lu]Lu-LW02060 and [^177^Lu]Lu-LW02080.

Target Organ	[^177^Lu]Lu-LW02060	[^177^Lu]Lu-LW02080
Adrenals	2.45 × 10^−2^	4.24 × 10^−3^
Brain	2.85 × 10^−4^	1.04 × 10^−4^
Esophagus	8.10 × 10^−4^	3.14 × 10^−4^
Eyes	5.72 × 10^−4^	2.45 × 10^−4^
Gallbladder wall	1.20 × 10^−3^	4.15 × 10^−4^
Left colon	2.59 × 10^−2^	5.58 × 10^−3^
Small intestine	7.60 × 10^−3^	3.97 × 10^−3^
Stomach wall	3.70 × 10^−3^	1.45 × 10^−3^
Right colon	1.35 × 10^−2^	2.98 × 10^−3^
Rectum	1.30 × 10^−2^	3.00 × 10^−3^
Heart	2.66 × 10^−3^	1.17 × 10^−3^
Kidneys	6.36 × 10^−2^	2.78 × 10^−2^
Liver	1.29 × 10^−2^	3.17 × 10^−3^
Lungs	5.31 × 10^−3^	2.11 × 10^−3^
Pancreas	5.08 × 10^−2^	1.69 × 10^−3^
Prostate	1.64 × 10^−3^	6.94 × 10^−4^
Salivary glands	5.91 × 10^−4^	2.52 × 10^−4^
Red marrow	7.68 × 10^−4^	3.23 × 10^−4^
Skeleton	1.04 × 10^−3^	4.73 × 10^−4^
Spleen	9.47 × 10^−3^	2.92 × 10^−3^
Testes	2.49 × 10^−3^	1.05 × 10^−3^
Thymus	6.92 × 10^−4^	2.85 × 10^−4^
Thyroid	6.38 × 10^−4^	2.68 × 10^−4^
Urinary bladder	1.50 × 10^−1^	6.51 × 10^−2^
Total body	2.55 × 10^−3^	9.77 × 10^−4^
Effective dose (mSv/MBq)	1.16 × 10^−2^	4.15 × 10^−3^

## Data Availability

The data presented in this study are available in the Supplementary Materials.

## Supplementary Materials

The following supporting information can be downloaded at https://www.mdpi.com/article/10.3390/ph18020234/s1: Detailed procedures for cell culture and synthetic procedures, results and characterizations for Fmoc-Leu(ψ)diF-Pro-OH (**5**) and the intermediates; Scheme S1: Synthesis of Fmoc-Leu(ψ)diF-Pro-OH (**5**); Table S1: HPLC purification conditions and MS characterizations of LW02060 and LW02080; Table S2: HPLC purification conditions and MS characterizations of Ga/Lu-LW02060 and Ga/Lu-LW02080; Table S3: HPLC conditions for purification and quality control of ^68^Ga/^177^Lu-labeled LW02060 and LW02080; Table S4: Biodistribution and uptake ratios of [^68^Ga]Ga-LW02060 and [^68^Ga]Ga-LW02080 in PC-3 tumor-bearing mice; Table S5: Biodistribution and tumor-to-organ uptake ratios of [^177^Lu]Lu-LW02060 in PC-3 tumor-bearing mice at 1, 4, 24, 72, and 120 h post-injection; Table S6: Biodistribution and tumor-to-organ uptake ratios of [^177^Lu]Lu-LW02080 in PC-3 tumor-bearing mice at 1, 4, 24, 72, and 120 h post-injection; Table S7: Radiation absorbed doses with the tumor sink effect correction for selected mouse organs/tissues and PC-3 tumor xenografts per unit of injected activity from [^177^Lu]Lu-LW02060 and [^177^Lu]Lu-LW02080; Table S8: Radiation absorbed radiation doses without the tumor sink effect correction for selected mouse organs/tissues per unit of injected activity from [^177^Lu]Lu-LW02060 and [^177^Lu]Lu-LW02080; Figure S1: The ^1^H NMR spectrum of Fmoc-diF-Pro-OtBu (**1**); Figure S2: The ^13^C NMR spectrum of Fmoc-diF-Pro-OtBu (**1**); Figure S3: The ^19^F NMR spectrum of Fmoc-diF-Pro-OtBu (**1**); Figure S4: The MS spectrum of Fmoc-diF-Pro-OtBu (**1**); Figure S5: The ^1^H NMR spectrum of diF-Pro-OtBu HCl salt (**2**); Figure S6: The ^13^C NMR spectrum of diF-Pro-OtBu HCl salt (**2**); Figure S7: The ^19^F NMR spectrum of diF-Pro-OtBu HCl salt (**2**); Figure S8: The MS spectrum of diF-Pro-OtBu HCl salt (**2**); Figure S9: The ^1^H NMR spectrum of Fmoc-Leu(ψ)diF-Pro-OtBu (**4**); Figure S10: The ^13^C NMR spectrum of Fmoc-Leu(ψ)diF-Pro-OtBu (**4**); Figure S11: The ^19^F NMR spectrum of Fmoc-Leu(ψ)diF-Pro-OtBu (**4**); Figure S12: The MS spectrum of Fmoc-Leu(ψ)diF-Pro-OtBu (**4**); Figure S13: The ^1^H NMR spectrum of Fmoc-Leu(ψ)diF-Pro-OH HCl salt (**5**); Figure S14: The ^13^C NMR spectrum of Fmoc-Leu(ψ)diF-Pro-OH HCl salt (**5**); Figure S15: The ^19^F NMR spectrum of Fmoc-Leu(ψ)diF-Pro-OH HCl salt (**5**); Figure S16: The MS spectrum of Fmoc-Leu(ψ)diF-Pro-OH HCl (**5**); Figure S17: The MS spectrum of LW02060; Figure S18: The MS spectrum of LW02080; Figure S19: The MS spectrum of Ga-LW02060; Figure S20: The MS spectrum of Lu-LW02060; Figure S21: The MS spectrum of Ga-LW02080; Figure S22: The MS spectrum of Lu-LW02080; Figure S23: A representative analytical radio-HPLC chromatogram of the purified [^68^Ga]Ga-LW02060; Figure S24: A representative analytical radio-HPLC chromatogram of the purified [^68^Ga]Ga-LW02080; Figure S25: A representative analytical radio-HPLC chromatogram of the purified [^177^Lu]Lu-LW02060; Figure S26: A representative analytical radio-HPLC chromatogram of the purified [^177^Lu]Lu-LW02080.

## References

[1] Jensen R., Battey J., Spindel E., Benya R. (2008). International Union of Pharmacology. LXVIII. Mammalian bombesin receptors: Nomenclature, distribution, pharmacology, signaling, and functions in normal and disease states. Pharmacol. Rev..

[2] Weber H.C. (2009). Regulation and signaling of human bombesin receptors and their biological effects. Curr. Opin. Endocrinol. Diabetes Obes..

[3] Cornelio D.B., Roesler R., Schwartsmann G. (2007). Gastrin-releasing peptide receptor as a molecular target in experimental anticancer therapy. Ann. Oncol..

[4] Markwalder R., Reubi J.C. (1999). Gastrin-releasing peptide receptors in the human prostate: Relation to neoplastic transformation. Cancer Res..

[5] Gugger M., Reubi J.C. (1999). Gastrin-releasing peptide receptors in non-neoplastic and neoplastic human breast. Am. J. Pathol..

[6] Preston S., Woodhouse L., Jones-Blackett S., Miller G., Primrose J. (1995). High-affinity binding sites for gastrin-releasing peptide on human colorectal cancer tissue but not uninvolved mucosa. Br. J. Cancer.

[7] Mattei J., Achcar R.D., Cano C.H., Macedo B.R., Meurer L., Batlle B.S., Groshong S.D., Kulczynski J.M., Roesler R., Dal Lago L. (2014). Gastrin-releasing peptide receptor expression in lung cancer. Arch. Pathol. Lab. Med..

[8] Shriver S.P., Bourdeau H.A., Gubish C.T., Tirpak D.L., Davis A.L.G., Luketich J.D., Siegfried J.M. (2000). Sex-specific expression of gastrin-releasing peptide receptor: Relationship to smoking history and risk of lung cancer. J. Natl. Cancer Inst..

[9] Kurth J., Krause B.J., Schwarzenböck S.M., Bergner C., Hakenberg O.W., Heuschkel M. (2020). First-in-human dosimetry of gastrin-releasing peptide receptor antagonist [^177^Lu]Lu-RM2: A radiopharmaceutical for the treatment of metastatic castration-resistant prostate cancer. Eur. J. Nucl. Med. Mol. Imaging.

[10] Nock B.A., Kaloudi A., Lymperis E., Giarika A., Kulkarni H.R., Klette I., Singh A., Krenning E.P., de Jong M., Maina T. (2017). Theranostic perspectives in prostate cancer with the gastrin-releasing peptide receptor antagonist NeoBOMB1: Preclinical and first clinical results. J. Nucl. Med..

[11] Varvarigou A., Bouziotis P., Zikos C., Scopinaro F., De Vincentis G. (2004). Gastrin-releasing peptide (GRP) analogues for cancer imaging. Cancer Biother. Radiopharm..

[12] Baum R., Prasad V., Mutloka N., Frischknecht M., Maecke H., Reubi J. (2007). Molecular imaging of bombesin receptors in various tumors by Ga-68 AMBA PET/CT: First results. J. Nucl. Med..

[13] Kähkönen E., Jambor I., Kemppainen J., Lehtiö K., Grönroos T.J., Kuisma A., Luoto P., Sipilä H.J., Tolvanen T., Alanen K. (2013). In vivo imaging of prostate cancer using [^68^Ga]-labeled bombesin analog BAY86-7548. Clin. Cancer Res..

[14] Marsouvanidis P.J., Maina T., Sallegger W., Krenning E.P., de Jong M., Nock B.A. (2013). ^99m^Tc radiotracers based on human GRP(18–27): Synthesis and comparative evaluation. J. Nucl. Med..

[15] Reile H., Cai R., Armatis P., Schally A. (1995). New antagonists of bombesin gastrin-releasing peptide with C-terminal Leu-psi-(CH_2_N)Tac-NH_2_. Int. J. Oncol..

[16] Cai R., Reile H., Armatis P., Schally A.V. (1994). Potent bombesin antagonists with C-terminal Leu-psi-(CH_2_N)Tac-NH_2_ or its derivatives. Proc. Natl. Acad. Sci. USA.

[17] Minamimoto R., Hancock S., Schneider B., Chin F.T., Jamali M., Loening A., Vasanawala S., Gambhir S.S., Iagaru A. (2016). Pilot comparison of ^68^Ga-RM2 PET and ^68^Ga-PSMA-11 PET in patients with biochemically recurrent prostate cancer. J. Nucl. Med..

[18] Chatalic K.L., Konijnenberg M., Nonnekens J., de Blois E., Hoeben S., de Ridder C., Brunel L., Fehrentz J.-A., Martinez J., van Gent D.C. (2016). In vivo stabilization of a gastrin-releasing peptide receptor antagonist enhances PET imaging and radionuclide therapy of prostate cancer in preclinical studies. Theranostics.

[19] Nock B.A., Maina T., Krenning E.P., de Jong M. (2014). “To serve and protect”: Enzyme inhibitors as radiopeptide escorts promote tumor targeting. J. Nucl. Med..

[20] Wang L., Zhang Z., Merkens H., Zeisler J., Zhang C., Roxin A., Tan R., Bénard F., Lin K.-S. (2022). ^68^Ga-labeled [Leu^13^ψThz1^4^]Bombesin(7–14) derivatives: Promising GRPR-targeting PET tracers with low pancreas uptake. Molecules.

[21] Wang L., Bratanovic I.J., Zhang Z., Kuo H.-T., Merkens H., Zeisler J., Zhang C., Tan R., Bénard F., Lin K.-S. (2023). ^68^Ga-Labeled [Thz^14^]Bombesin(7–14) analogs: Promising GRPR-targeting agonist PET tracers with low pancreas uptake. Molecules.

[22] Wang L., Kuo H.-T., Zhang Z., Zhang C., Chen C.-C., Chapple D., Wilson R., Colpo N., Bénard F., Lin K.-S. (2024). Unnatural amino acid substitutions to improve in vivo stability and tumor uptake of ^68^Ga-labeled GRPR-targeted TacBOMB2 derivatives for cancer imaging with positron emission tomography. EJNMMI Radiopharm. Chem..

[23] Wang L., Chen C.-C., Zhang Z., Kuo H.-T., Zhang C., Colpo N., Merkens H., Bénard F., Lin K.-S. (2024). Synthesis and evaluation of novel ^68^Ga-Labeled [D-Phe^6^,Leu^13^ψThz^14^]bombesin(6–14) analogs for cancer imaging with positron emission tomography. Pharmaceuticals.

[24] Wang L., Kuo H.-T., Chapple D.E., Chen C.-C., Kurkowska S., Colpo N., Uribe C., Bénard F., Lin K.-S. (2024). Synthesis and evaluation of ^68^Ga- and ^177^Lu-Labeled [Pro^14^]bombesin(8–14) derivatives for detection and radioligand therapy of gastrin-releasing peptide receptor-expressing cancer. Mol. Pharm..

[25] Jeffries B., Wang Z., Felstead H.R., Le Questel J.-Y., Scott J.S., Chiarparin E., Graton J., Linclau B. (2020). Systematic investigation of lipophilicity modulation by aliphatic fluorination motifs. J. Med. Chem..

[26] Mykhailiuk P.K. (2022). Fluorine-containing prolines: Synthetic strategies, applications, and opportunities. J. Org. Chem..

[27] Jansen K., Heirbaut L., Verkerk R., Cheng J.D., Joossens J., Cos P., Maes L., Lambeir A.-M., De Meester I., Augustyns K. (2014). Extended structure–activity relationship and pharmacokinetic investigation of (4-Quinolinoyl)glycyl-2-cyanopyrrolidine Inhibitors of Fibroblast Activation Protein (FAP). J. Med. Chem..

[28] Li M., Younis M.H., Zhang Y., Cai W., Lan X. (2022). Clinical summary of fibroblast activation protein inhibitor-based radiopharmaceuticals: Cancer and beyond. Eur. J. Nucl. Med. Mol. Imaging.

[29] Mansi R., Fleischmann A., Mäcke H.R., Reubi J.C. (2013). Targeting GRPR in urological cancers—From basic research to clinical application. Nat. Rev. Urol..

[30] Yang M., Gao H., Zhou Y., Ma Y., Quan Q., Lang L., Chen K., Niu G., Yan Y., Chen X. (2011). ^18^F-labeled GRPR agonists and antagonists: A comparative study in prostate cancer imaging. Theranostics.

[31] Fani M., Braun F., Waser B., Beetschen K., Cescato R., Erchegyi J., Rivier J.E., Weber W.A., Maecke H.R., Reubi J.C. (2012). Unexpected sensitivity of sst_2_ antagonists to N-terminal radiometal modifications. J. Nucl. Med..

[32] Virtanen P., Gommers R., Oliphant T.E., Haberland M., Reddy T., Cournapeau D., Burovski E., Peterson P., Weckesser W., Bright J. (2020). SciPy 1.0: Fundamental algorithms for scientific computing in Python. Nat. Methods.

[33] Keenan M.A., Stabin M.G., Segars W.P., Fernald M.J. (2010). RADAR realistic animal model series for dose assessment. J. Nucl. Med..

[34] Cicone F., Denoël T., Gnesin S., Riggi N., Irving M., Jakka G., Schaefer N., Viertl D., Coukos G., Prior J.O. (2020). Preclinical evaluation and dosimetry of [^111^In]CHX-DTPA-scFv78-Fc targeting endosialin/tumor endothelial marker 1 (TEM1). Mol. Imaging Biol..

[35] Stabin M.G., Sparks R.B., Crowe E. (2005). OLINDA/EXM: The second-generation personal computer software for internal dose assessment in nuclear medicine. J. Nucl. Med..

[36] Kirschner A., Ice R., Beierwaltes W. (1973). Radiation dosimetry of ^131^I-19-iodocholesterol. J. Nucl. Med..

[37] Stabin M.G., Xu X.G., Emmons M.A., Segars W.P., Shi C., Fernald M.J. (2012). RADAR reference adult, pediatric, and pregnant female phantom series for internal and external dosimetry. J. Nucl. Med..

[38] Stabin M.G., Konijnenberg M.W. (2000). Re-evaluation of absorbed fractions for photons and electrons in spheres of various sizes. J. Nucl. Med..

