# Supplementary material for: Synthesis and Evaluation of 68Ga- and 177Lu-Labeled [diF-Pro14]Bombesin(6−14) Analogs for Detection and Radioligand Therapy of Gastrin-Releasing Peptide Receptor-Expressing Cancer
Source: Pharmaceuticals (Basel). 2025 Feb 8;18(2):234. doi: 10.3390/ph18020234 (PMC11859184; doi:10.3390/ph18020234)
Supplement: Supplementary file 1 [file pharmaceuticals-18-00234-s001.zip › pharmaceuticals-3460050-supplementary.pdf]

SUPPLEMENTARY INFORMATION

Synthesis of Fmoc-Leu(ψ)diF-Pro-OH (5)

Compound **5** was synthesized following the reaction steps depicted in Scheme S1.

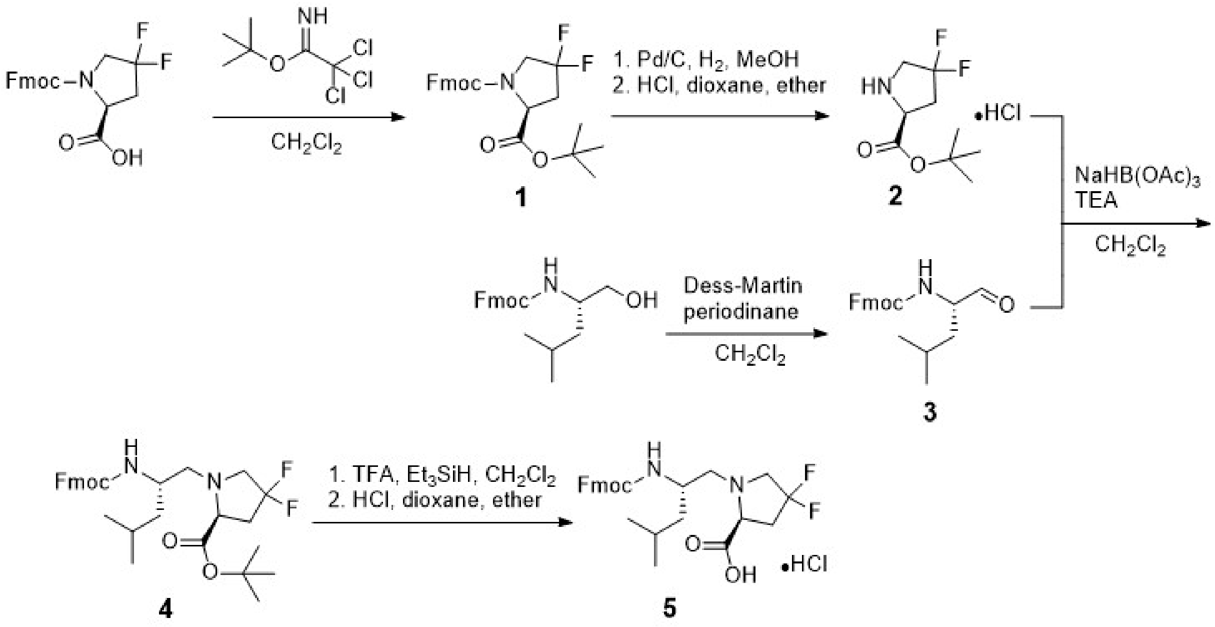

Scheme S1: Synthesis of Fmoc-Leu(ψ)diF-Pro-OH (**5**).

Synthesis of Fmoc-diF-Pro-OtBu (**1**): *t*-Butyl trichloroacetimidate (4.37 g, 20 mmol) was added to a solution of (2*S*)-Fmoc-4,4-difluoro-pyrrolidine-2-carboxylic acid (Fmoc-diF-Pro-OH, 3.73 g, 10 mmol) in dichloromethane (30 mL), and the resulting solution was stirred at room temperature for 22 h. After evaporation, the residue was purified by flash column chromatography eluted with 1:2 diethyl ether/hexanes to obtain 3.76 g (88%) of compound **1** as a colorless thick oil. ESI-MS: m/z calculated for $[M+Na]^+$ of **1** $C_{24}H_{25}F_2NO_4$ 452.2; found 452.2. ^1H NMR (300 MHz, $CDCl_3$) δ 7.77 (d, *J* = 7.5 Hz, 2H), 7.65 – 7.52 (m, 2H), 7.41 (t, *J* = 7.4 Hz, 2H), 7.32 (td, *J* = 7.5, 2.3 Hz, 2H), 4.56 – 4.31 (m, 3H), 4.23 (dt, *J* = 20.7, 7.1 Hz, 1H), 4.04 – 3.79 (m, 2H), 2.83 – 2.38 (m, 1H), 1.50 – 1.41 (m, 9H). ^{13}C NMR (75 MHz, $CDCl_3$) δ 169.58 (d, *J* = 14.7 Hz), 154.18, 144.86 – 142.88 (m), 141.45, 127.96, 127.26, 125.16, 120.18, 82.82 (d, *J* = 13.1 Hz), 68.14 (d, *J* = 13.6 Hz), 57.92, 53.64 (d, *J* = 23.4 Hz), 47.29 (d, *J* = 9.3 Hz), 39.78 – 37.35 (m), 28.04. ^{19}F NMR (282 MHz, $CDCl_3$) δ -96.90 – -100.27 (m).

Synthesis of diF-Pro-OtBu HCl salt (**2**): A solution of compound **1** (3.72 g, 8.7 mmol) and palladium on charcoal (10%, 300 mg) in methanol (80 mL) was hydrogenated using a balloon. After 2 days, the reaction mixture was filtered through celite, and the filtrate was evaporated under reduced pressure. The residue was dissolved in diethyl ether (200 mL). After the addition of HCl (4 N in 1,4-dioxane, 5 mL), the solution was stirred for 10 min. The resulting precipitate was filtered and dried under vacuum to obtain 1.78 g (84%) of compound **2** as a white solid. ESI-MS: m/z calculated for $[M+H]^+$ of **2** $C_9H_{15}F_2NO_2$ 208.1; found 208.2. ^1H NMR (300 MHz, DMSO-d_6) δ 4.68 (t, *J* = 8.2 Hz, 1H), 3.86 – 3.58 (m, 2H), 2.95 – 2.54 (m, 2H), 1.47 (s,

9H).^{13}C NMR (75 MHz, DMSO-d_6) δ 165.97, 127.10 (t, J = 248.4 Hz), 84.05, 57.29 (t, J = 4.1 Hz), 50.51 (t, J = 33.8 Hz), 36.52 (t, J = 25.2 Hz), 27.45. ^{19}F NMR (282 MHz, DMSO-d_6) δ -94.24 (d, J = 234.6 Hz), -96.07 (d, J = 234.5 Hz).

Synthesis of Fmoc-Leu(ψ)diF-Pro-OtBu (**4**): A solution of Fmoc-leucinol (2.20 g, 6.5 mmol) in dichloromethane (40 mL) cooled in an ice/water bath was added Dess-Martin periodinane (3.44 g, 8.1 mmol). After stirring for 22 h, a solution of sodium thiosulfate (4.5 g) in saturated sodium bicarbonate aqueous solution (45 mL) was added, and the resulting solution was stirred for 30 min. After separating the two phases, the aqueous phase was extracted with dichloromethane (50 mL). The organic phases were combined, dried over anhydrous magnesium sulfate, and evaporated under reduced pressure to yield 2.33 g of crude aldehyde **3** as a yellowish solid.

A solution of the crude aldehyde **3** (2.33 g), diF-Pro-OtBu HCl salt (**2**, 1.70 g, 7.0 mmol), triethylamine (809 mg, 8 mmol) and sodium triacetoxyborohydride (3.18 g, 15 mmol) in dichloromethane (40 mL) was stirred for 2 days. After addition of 100 mL of saturated sodium bicarbonate aqueous solution, the resulting mixture was stirred for 10 min. The phases were separated and the aqueous phase was extracted with dichloromethane (50 mL). The organic phases were combined, dried over anhydrous magnesium sulfate, and evaporated under reduced pressure. The residue was purified by flash column chromatography eluted with 1:4 ethyl acetate/hexanes to obtain 2.51 g of compound **4** as a thick colorless oil. The yield is 73% over 2 steps. ESI-MS: m/z calculated for $[M+H]^+$ of **4** $C_{30}H_{38}F_2N_2O_4$ 529.3; found 529.2. ^1H NMR (300 MHz, $CDCl_3$) δ 7.81 – 7.71 (m, 2H), 7.61 (d, J = 7.3 Hz, 2H), 7.40 (td, J = 7.5, 1.3 Hz, 2H), 7.31 (td, J = 7.4, 1.3 Hz, 2H), 5.07 (d, J = 8.2 Hz, 1H), 4.39 (d, J = 6.4 Hz, 2H), 4.21 (t, J = 6.6 Hz, 1H), 3.71 (s, 1H), 3.47 (d, J = 9.8 Hz, 2H), 3.05 – 2.93 (m, 1H), 2.77 (d, J = 12.4 Hz, 1H), 2.61 (d, J = 12.5 Hz, 1H), 2.55 – 2.29 (m, 2H), 1.58 (s, 2H), 1.46 (s, 9H), 1.31 – 1.21 (m, 1H), 1.01 – 0.85 (m, 6H). ^{13}C NMR (75 MHz, $CDCl_3$) δ 156.24, 141.48, 127.78, 125.25, 120.09, 58.80, 48.92, 47.53, 39.51 (t, J = 25.7 Hz), 28.18, 24.91. ^{19}F NMR (282 MHz, $CDCl_3$) δ -92.81 (d, J = 229.7 Hz), -95.54 (d, J = 229.6 Hz).

Synthesis of Fmoc-Leu(ψ)diF-Pro-OH HCl salt (**5**): A solution of compound **4** (2.48 g, 4.7 mmol) and triethylsilane (1.37 g, 11.8 mmol) in dichloromethane (25 mL) was added trifluoroacetic acid (75 mL). The resulting solution was stirred at room temperature for 3 h. The volatile solvents were evaporated under reduced pressure, and the residue was dissolved in diethyl ether (200 mL). HCl (4 N in 1,4-dioxane, 4 mL) was added to the ethereal solution, and the resulting solution was stirred for 5 min. The formed precipitate was filtered and dried under vacuum to obtain 1.87 g (78%) of compound **5** as a white solid. ESI-MS: m/z calculated for $[M+H]^+$ of **5** $C_{26}H_{30}F_2N_2O_4$ 473.2; found 473.2. ^1H NMR (300 MHz, $CDCl_3$) δ 7.76 (d, J = 7.5 Hz, 2H), 7.58 (d, J = 7.6 Hz, 2H), 7.40 (t, J = 7.4 Hz, 2H), 7.31 (t, J = 7.5 Hz, 2H), 4.95 (d, J = 8.4 Hz, 1H), 4.50 (d, J = 6.4 Hz, 2H), 4.19 (t, J = 5.8 Hz, 1H), 3.92 – 3.75 (m, 2H), 3.58 (d, J = 12.2 Hz, 1H), 3.15 (d, J = 12.2 Hz, 1H), 2.99 – 2.66 (m, 1H), 2.54 (dd, J = 14.6, 7.2 Hz, 1H), 1.59 (s, 1H), 1.28 (d, J = 13.6 Hz, 2H), 0.88 (t, J = 6.6 Hz, 6H). ^{13}C NMR (75 MHz, $CDCl_3$) δ 157.0, 143.8, 141.5, 128.0, 127.3, 125.1, 120.2, 66.9, 65.8, 60.7, 48.2, 47.3, 41.4, 37.7, 29.8, 24.7, 23.1, 21.7. ^{19}F NMR (282 MHz, $CDCl_3$) δ -95.04 (dd, J = 549.5, 239.7 Hz), -96.45 (dd, J = 807.1, 239.7 Hz).

Cell Culture

The PC-3 cells obtained from ATCC (via Cedarlane, Burlington, Canada) were cultured in RPMI 1640 medium (Life Technologies Corporations) supplemented with 10% FBS, penicillin (100 U/mL) and

streptomycin (100 µg/mL) at 37 °C in a Panasonic Healthcare (Tokyo, Japan) MCO-19AIC humidified incubator containing 5% CO_2. The cells were confirmed pathogen-free via IMPACT Rodent Pathogen Test (IDEXX BioAnalytics, Columbia, MO, USA). Cells grown to 80-90% confluence were washed with sterile DPBS (pH 7.4) and collected after 1-min trypsinization at 37 °C. The cell concentration was measured in duplicate using a Moxi mini automated cell counter (ORFLO Technologies, Ketchum, ID, USA).

Table S1: HPLC purification conditions and MS characterizations of LW02060 and LW02080.

Compound name	HPLC conditions	Retention time (min)	Yield (%)	Calculated mass (m/z)	Found (m/z)
LW02060	25% CH_3CN and 0.1% TFA in H_2O	13.6	23	$[M+2H]^{2+}$ 822.4	$[M+2H]^{2+}$ 822.8
LW02080	25% CH_3CN and 0.1% TFA in H_2O	12.0	39	$[M+2H]^{2+}$ 808.4	$[M+2H]^{2+}$ 808.8

Table S2: HPLC purification conditions and MS characterizations of Ga/Lu-LW02060 and Ga/Lu-LW02080.

Compound name	HPLC conditions	Retention time (min)	Yield (%)	Calculated mass (m/z)	Found (m/z)
Ga-LW02060	25% CH_3CN and 0.1% TFA in H_2O	15.9	87	$[M+2H]^{2+}$ 855.9	$[M+2H]^{2+}$ 855.8
Lu-LW02060	25% CH_3CN and 0.1% TFA in H_2O	16.0	93	$[M+2H]^{2+}$ 908.4	$[M+2H]^{2+}$ 908.7
Ga-LW02080	25% CH_3CN and 0.1% TFA in H_2O	15.4	87	$[M+2H]^{2+}$ 841.9	$[M+2H]^{2+}$ 841.9
Lu-LW02080	25% CH_3CN and 0.1% TFA in H_2O	14.0	90	$[M+2H]^{2+}$ 894.4	$[M+2H]^{2+}$ 894.7

Table S3: HPLC conditions for purification and quality control of ^{68}Ga/^{177}Lu-labeled LW02060 and LW02080.

Compound name	HPLC conditions		Retention time (min)
[^{68}Ga]Ga-LW02060	Semi-prep	16% CH_3CN and 0.1% FA in H_2O; flow rate 4.5 mL/min	11.6
	QC	23% CH_3CN and 0.1% FA in H_2O; flow rate 2.0 mL/min	8.7
[^{177}Lu]Lu-LW02060	Semi-prep	20% CH_3CN and 0.1% FA in H_2O; flow rate 4.5 mL/min	12.4
	QC	21% CH_3CN and 0.1% FA in H_2O; flow rate 2 mL/min	8.4
[^{68}Ga]Ga-LW02080	Semi-prep	20% CH_3CN and 0.1% FA in H_2O; flow rate 4.5 mL/min	11.7
	QC	21% CH_3CN and 0.1% FA in H_2O; flow rate 2.0 mL/min	8.1
[^{177}Lu]Lu-LW02080	Semi-prep	20% CH_3CN and 0.1% TFA in H_2O; flow rate 4.5 mL/min	15.7
	QC	22.5% CH_3CN and 0.1% FA in H_2O; flow rate 2.0 mL/min	7.7

Table S4: Biodistribution (mean ± SD, n = 4) and uptake ratios of [^{68}Ga]Ga-LW02060 and [^{68}Ga]Ga-LW02080 in PC-3 tumor-bearing mice. The mice in the blocked group were co-injected with 100 µg of [D-Phe^{6},Leu-NHEt^{13},des-Met^{14}]Bombesin(6-14) for [^{68}Ga]Ga-LW02060, and 100 µg of nonradioactive standard for [^{68}Ga]Ga-LW02080. *,**,*** indicate $p < 0.05$, <0.01, and < 0.001, respectively.

Tissue (%ID/g)	[^{68}Ga]Ga-LW02060		[^{68}Ga]Ga-LW02080	
	1 h	1 h blocked	1 h	1 h blocked
Blood	0.54 ± 0.03	1.71 ± 0.44*	0.33 ± 0.08	2.02 ± 0.70**
Fat	0.05 ± 0.02	0.27 ± 0.10**	0.03 ± 0.01	0.25 ± 0.04**
Testes	0.20 ± 0.06	0.76 ± 0.29**	0.12 ± 0.03	0.63 ± 0.20**
Small intestine	0.96 ± 0.20	0.72 ± 0.02**	0.24 ± 0.07	1.04 ± 0.48*
Large intestine	0.61 ± 0.10	0.54 ± 0.13	0.30 ± 0.09	0.33 ± 0.10
Spleen	0.24 ± 0.10	0.53 ± 0.15*	0.18 ± 0.05	0.56 ± 0.17**
Pancreas	3.12 ± 0.89	2.25 ± 0.59	0.38 ± 0.04	0.51 ± 0.18*
Stomach	0.77 ± 0.29	0.38 ± 0.06	0.08 ± 0.04	0.38 ± 0.20**
Liver	0.37 ± 0.08	1.11 ± 0.43*	0.31 ± 0.07	1.05 ± 0.39***
Adrenal glands	0.66 ± 0.16	0.67 ± 0.03	0.39 ± 0.00	0.69 ± 0.05***
Kidneys	3.58 ± 1.14	11.3 ± 0.97***	2.42 ± 0.81	11.2 ± 1.02**
Heart	0.20 ± 0.05	0.52 ± 0.14**	0.11 ± 0.03	0.66 ± 0.18**
Lungs	0.61 ± 0.15	1.52 ± 0.39**	0.28 ± 0.05	1.52 ± 0.44***
PC-3 tumor	16.8 ± 2.70	6.11 ± 0.42***	7.36 ± 1.13	1.71 ± 0.39***
Bone	0.10 ± 0.04	0.29 ± 0.07**	0.11 ± 0.02	0.31 ± 0.06***
Muscle	0.12 ± 0.03	0.35 ± 0.08**	0.06 ± 0.02	0.53 ± 0.06*
Brain	0.02 ± 0.01	0.05 ± 0.01**	0.01 ± 0.00	0.06 ± 0.03*
Tumor to Normal Tissue Ratios				
Tumor/bone	179 ± 46.9	22.3 ± 8.24**	69.8 ± 13.7	5.52 ± 0.68***
Tumor/muscle	141 ± 19.0	17.7 ± 3.85***	117 ± 18.6	3.61 ± 0.14***
Tumor/blood	29.1 ± 3.62	3.72 ± 0.74***	22.7 ± 4.13	0.87 ± 0.15***
Tumor/kidney	4.86 ± 0.76	0.54 ± 0.03***	3.18 ± 0.75	0.14 ± 0.03**
Tumor/pancreas	5.52 ± 0.78	2.87 ± 0.84**	18.4 ± 2.86	3.47 ± 0.64***

Table S5: Biodistribution (mean ± SD, n = 5) and tumor-to-organ uptake ratios of [^{177}Lu]Lu-LW02060 in PC-3 tumor-bearing mice at 1, 4, 24, 72, and 120 h post-injection. The mice in the blocked group were co-injected with 100 µg of [D-Phe^{6},Leu-NHEt^{13},des-Met^{14}]Bombesin(6-14) per mouse. *,**,*** indicate p < 0.05, <0.01, and < 0.001, respectively.

Tissue (%ID/g)	[^{177}Lu]Lu-LW02060					
	1 h	4 h	24 h	72 h	120 h	1 h blocked
Blood	0.59 ± 0.18	0.06 ± 0.02	0.01 ± 0.00	0.00 ± 0.00	0.00 ± 0.00	0.43 ± 0.23
Fat	0.08 ± 0.02	0.02 ± 0.01	0.01 ± 0.00	0.00 ± 0.00	0.01 ± 0.01	0.06 ± 0.03
Testes	0.18 ± 0.03	0.04 ± 0.01	0.02 ± 0.00	0.02 ± 0.01	0.01 ± 0.01	0.14 ± 0.05
Small intestine	0.67 ± 0.16	0.26 ± 0.04	0.13 ± 0.01	0.04 ± 0.01	0.02 ± 0.01	0.23 ± 0.06***
Large intestine	0.49 ± 0.31	0.68 ± 0.12	0.43 ± 0.13	0.32 ± 0.14	0.05 ± 0.02	0.22 ± 0.04
Spleen	0.46 ± 0.20	0.13 ± 0.03	0.09 ± 0.02	0.07 ± 0.02	0.05 ± 0.01	0.20 ± 0.06**
Pancreas	2.64 ± 0.63	1.71 ± 0.08	1.04 ± 0.19	0.35 ± 0.08	0.11 ± 0.01	0.51 ± 0.12***
Stomach	0.29 ± 0.06	1.20 ± 1.55	0.12 ± 0.05	0.07 ± 0.02	0.03 ± 0.01	0.08 ± 0.03***
Liver	0.47 ± 0.19	0.27 ± 0.06	0.14 ± 0.07	0.06 ± 0.01	0.07 ± 0.02	0.27 ± 0.03
Adrenal glands	0.46 ± 0.15	0.33 ± 0.15	0.36 ± 0.30	0.13 ± 0.07	0.10 ± 0.02	0.27 ± 0.06
Kidneys	3.62 ± 0.86	2.85 ± 0.29	1.06 ± 0.22	0.33 ± 0.03	0.15 ± 0.04	3.80 ± 0.85
Heart	0.19 ± 0.05	0.06 ± 0.02	0.02 ± 0.00	0.01 ± 0.00	0.01 ± 0.00	0.13 ± 0.05
Lungs	0.57 ± 0.18	0.15 ± 0.04	0.06 ± 0.02	0.03 ± 0.01	0.02 ± 0.00	0.54 ± 0.17
PC-3 tumor	9.59 ± 3.37	8.38 ± 0.19	5.78 ± 0.40	2.74 ± 0.85	2.19 ± 0.59	3.25 ± 0.44**
Bone	0.23 ± 0.06	0.10 ± 0.08	0.02 ± 0.01	0.02 ± 0.00	0.02 ± 0.00	0.12 ± 0.04*
Muscle	0.13 ± 0.05	0.05 ± 0.01	0.01 ± 0.00	0.01 ± 0.00	0.00 ± 0.00	0.15 ± 0.05
Brain	0.02 ± 0.01	0.01 ± 0.00	0.00 ± 0.00	0.00 ± 0.00	0.00 ± 0.00	0.02 ± 0.01
Tumor to Normal Tissue Ratios						
Tumor/muscle	78.3 ± 13.0	196 ± 53.1	520 ± 113	522 ± 189	744 ± 179	23.2 ± 5.20***
Tumor/blood	16.1 ± 2.20	144 ± 33.5	813 ± 203	1125 ± 440	1626 ± 548	6.37 ± 1.92***
Tumor/kidney	2.65 ± 0.64	2.96 ± 0.30	5.67 ± 1.43	8.43 ± 2.89	14.1 ± 4.53	0.89 ± 0.22***
Tumor/pancreas	3.57 ± 0.42	4.91 ± 0.14	5.70 ± 0.81	8.37 ± 3.35	17.1 ± 2.10	6.72 ± 2.24*

Table S6: Biodistribution (mean ± SD, n = 5) and tumor-to-organ uptake ratios of [^{177}Lu]Lu-LW02080 in PC-3 tumor-bearing mice at 1, 4, 24, 72, and 120 h post-injection. The mice in the blocked group were co-injected with 100 µg of [D-Phe^{6},Leu-NHEt^{13},des-Met^{14}]Bombesin(6-14) per mouse. *,**,*** indicate $p <$ 0.05, <0.01, and < 0.001, respectively

Tissue (%ID/g)	[^{177}Lu]Lu-LW02080					
	1 h	4 h	24 h	72 h	120 h	1 h blocked
Blood	0.37 ± 0.14	0.02 ± 0.00	0.01 ± 0.01	0.00 ± 0.00	0.00 ± 0.00	0.74 ± 0.18*
Fat	0.04 ± 0.02	0.01 ± 0.01	0.01 ± 0.00	0.00 ± 0.00	0.00 ± 0.00	0.09 ± 0.04*
Testes	0.20 ± 0.04	0.03 ± 0.01	0.03 ± 0.01	0.01 ± 0.00	0.01 ± 0.00	0.22 ± 0.09
Small intestine	0.28 ± 0.03	0.07 ± 0.03	0.03 ± 0.01	0.01 ± 0.00	0.01 ± 0.00	0.48 ± 0.18*
Large intestine	0.17 ± 0.09	0.34 ± 0.04	0.15 ± 0.06	0.06 ± 0.02	0.02 ± 0.01	0.21 ± 0.09
Spleen	0.14 ± 0.04	0.07 ± 0.01	0.07 ± 0.02	0.04 ± 0.01	0.03 ± 0.00	0.16 ± 0.05
Pancreas	0.39 ± 0.08	0.06 ± 0.02	0.04 ± 0.01	0.01 ± 0.00	0.01 ± 0.00	0.34 ± 0.09
Stomach	0.08 ± 0.04	0.02 ± 0.00	0.07 ± 0.04	0.02 ± 0.01	0.01 ± 0.01	0.26 ± 0.14*
Liver	0.27 ± 0.10	0.20 ± 0.08	0.09 ± 0.01	0.04 ± 0.01	0.03 ± 0.01	0.33 ± 0.08
Adrenal glands	0.18 ± 0.05	0.17 ± 0.09	0.12 ± 0.05	0.02 ± 0.01	0.03 ± 0.02	0.23 ± 0.06
Kidneys	2.65 ± 0.48	1.88 ± 0.35	0.89 ± 0.28	0.26 ± 0.08	0.12 ± 0.03	4.17 ± 0.95*
Heart	0.13 ± 0.05	0.03 ± 0.01	0.02 ± 0.01	0.01 ± 0.00	0.01 ± 0.01	0.23 ± 0.05*
Lungs	0.43 ± 0.04	0.13 ± 0.08	0.05 ± 0.03	0.02 ± 0.00	0.05 ± 0.03	0.45 ± 0.22
PC-3 tumor	5.67 ± 1.02	2.96 ± 0.37	1.12 ± 0.20	0.55 ± 0.15	0.32 ± 0.06	2.02 ± 0.44***
Bone	0.06 ± 0.01	0.01 ± 0.00	0.02 ± 0.01	0.01 ± 0.01	0.01 ± 0.01	0.07 ± 0.04
Muscle	0.06 ± 0.01	0.01 ± 0.00	0.01 ± 0.00	0.00 ± 0.00	0.00 ± 0.00	0.15 ± 0.04**
Brain	0.02 ± 0.00	0.01 ± 0.00	0.00 ± 0.00	0.00 ± 0.00	0.00 ± 0.00	0.02 ± 0.01
Tumor to Normal Tissue Ratios						
Tumor/muscle	90.0 ± 17.3	275 ± 35.0	149 ± 50.5	356 ± 110	214 ± 77.9	14.6 ± 2.50***
Tumor/blood	16.9 ± 5.57	136 ± 14.7	365 ± 251	1297 ± 703	529 ± 380	3.06 ± 0.99**
Tumor/kidney	2.17 ± 0.49	1.61 ± 0.30	1.34 ± 0.38	2.17 ± 0.33	2.77 ± 0.65	0.52 ± 0.08***
Tumor/pancreas	14.8 ± 2.39	48.1 ± 11.3	26.5 ± 4.82	46.8 ± 13.9	51.5 ± 13.9	6.53 ± 0.76***

Table S7. Radiation absorbed doses with the tumor sink effect correction for selected mouse organs/tissues and PC-3 tumor xenografts per unit of injected activity (mGy/MBq) from [^{177}Lu]Lu-LW02060 and [^{177}Lu]Lu-LW02080.

Target Organ	[^{177}Lu]Lu-LW02060	[^{177}Lu]Lu-LW02080
Brain	2.39	1.01
Large intestine	62.9	13.6
Small intestine	19.5	10.2
Stomach wall	27.5	10.2
Heart	6.10	2.66
Kidneys	148	64.4
Liver	31.2	7.92
Lungs	13.0	5.21
Pancreas	118	5.03
Skeleton	7.96	5.77
Spleen	24.2	7.29
Testes	7.95	3.38
Thyroid	1.79	0.781
Bladder	1510	655
Total body	18.0	6.71
PC-3 tumor (1 g)	272	47.0

Table S8. Radiation absorbed doses without the tumor sink effect correction for selected mouse organs/tissues per unit of injected activity (mGy/MBq) from [^{177}Lu]Lu-LW02060 and [^{177}Lu]Lu-LW02080.

Target Organ	[^{177}Lu]Lu-LW02060	[^{177}Lu]Lu-LW02080
Brain	1.16	0.77
Large intestine	30.6	10.4
Small intestine	9.49	7.74
Stomach wall	13.4	7.79
Heart	2.96	2.02
Kidneys	72.1	49.0
Liver	15.2	6.03
Lungs	6.31	3.96
Pancreas	57.3	3.83
Skeleton	3.87	4.39
Spleen	11.8	5.55
Testes	3.86	2.57
Thyroid	0.869	0.594
Bladder	734	499
Total body	8.77	5.11

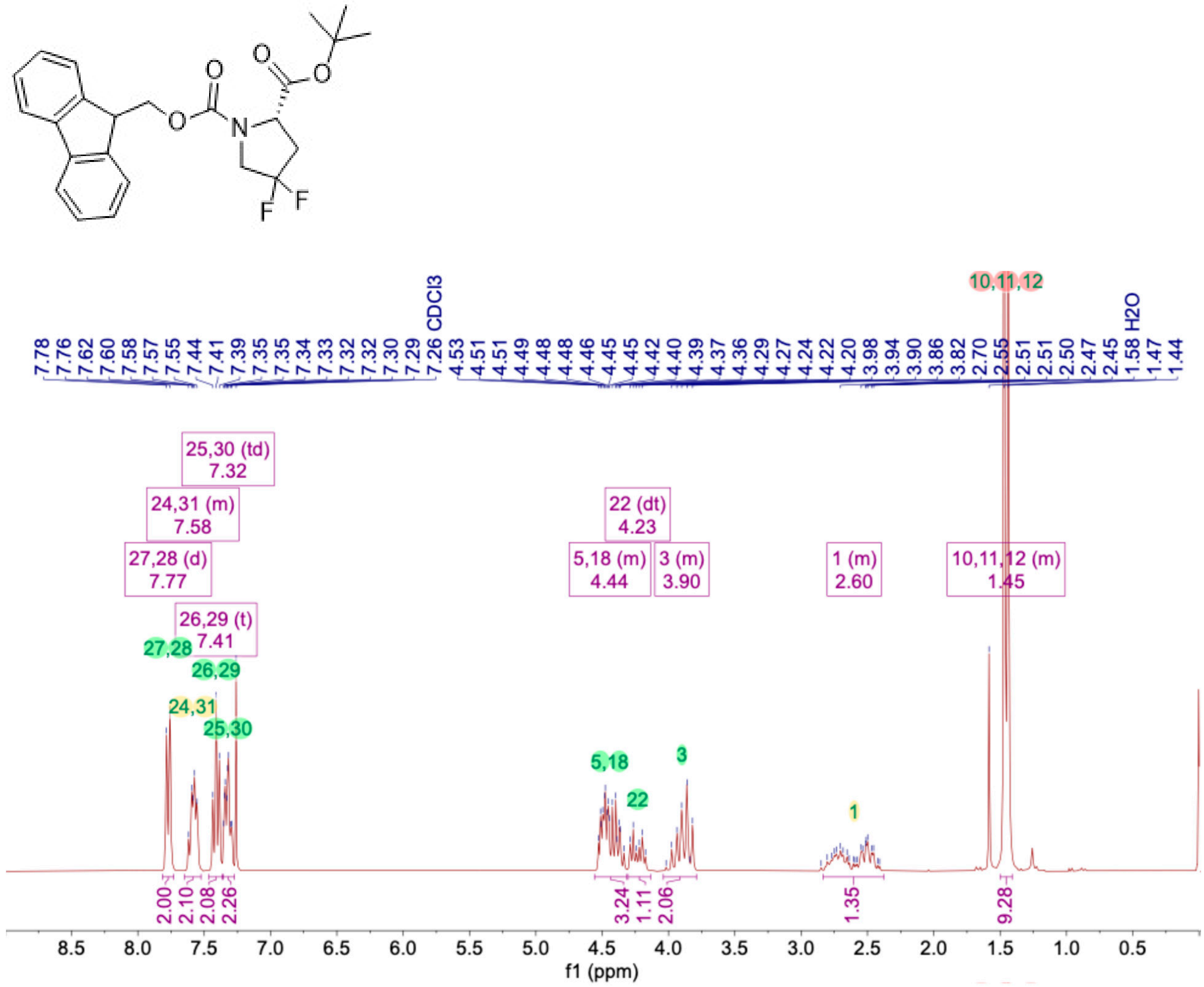

Figure S1. The ^{1}H NMR spectrum of Fmoc-diF-Pro-OtBu (**1**).

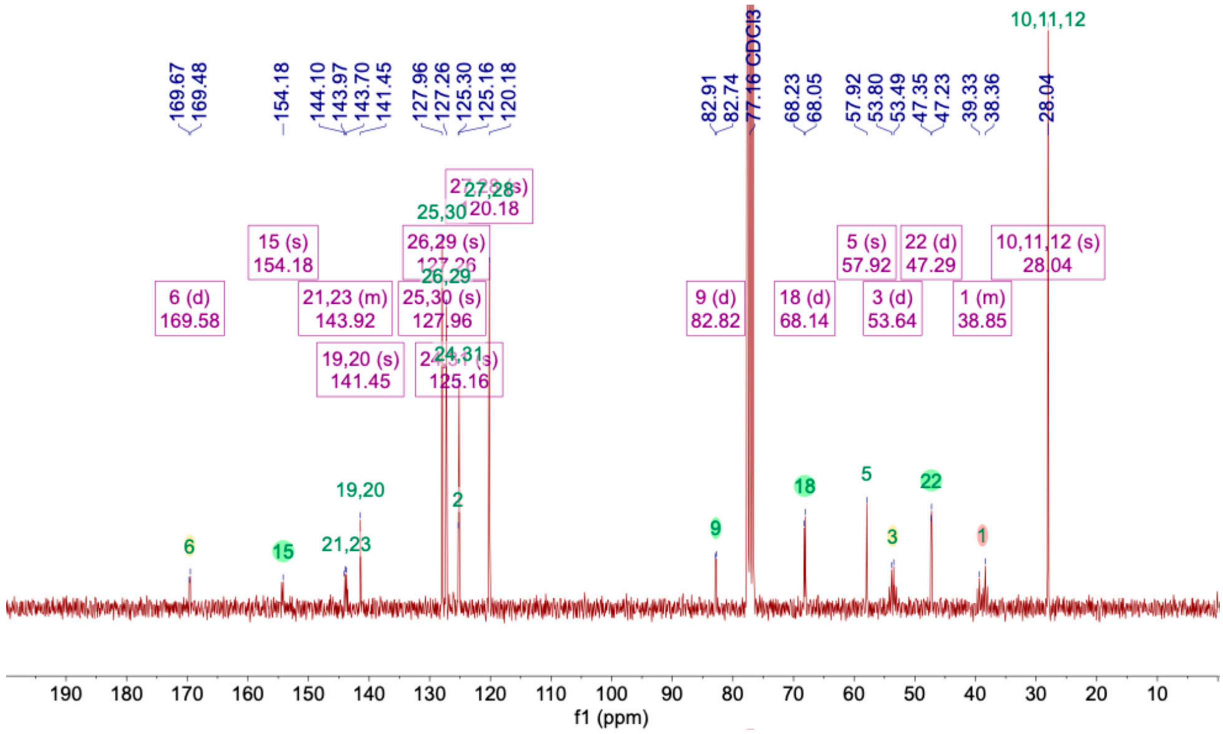

Figure S2. The ^{13}C NMR spectrum of Fmoc-diF-Pro-OtBu (**1**).

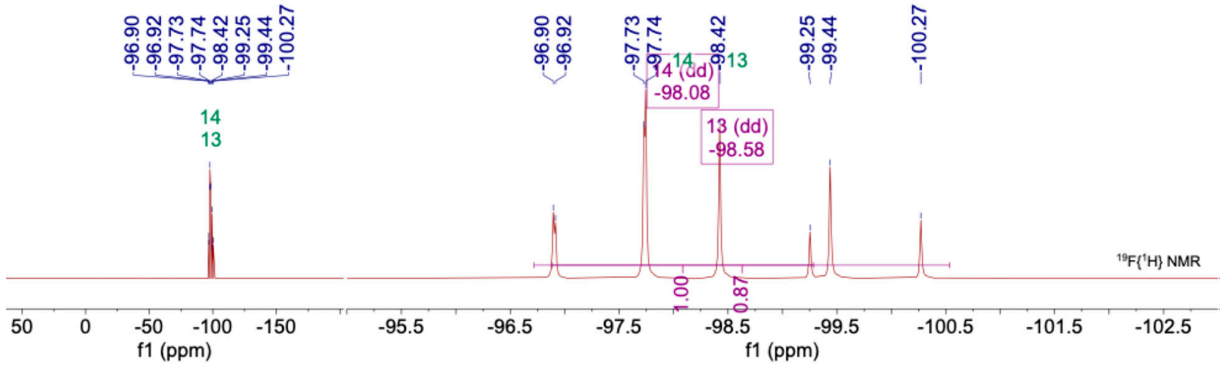

Figure S3. The ^{19}F NMR spectrum of Fmoc-diF-Pro-OtBu (**1**).

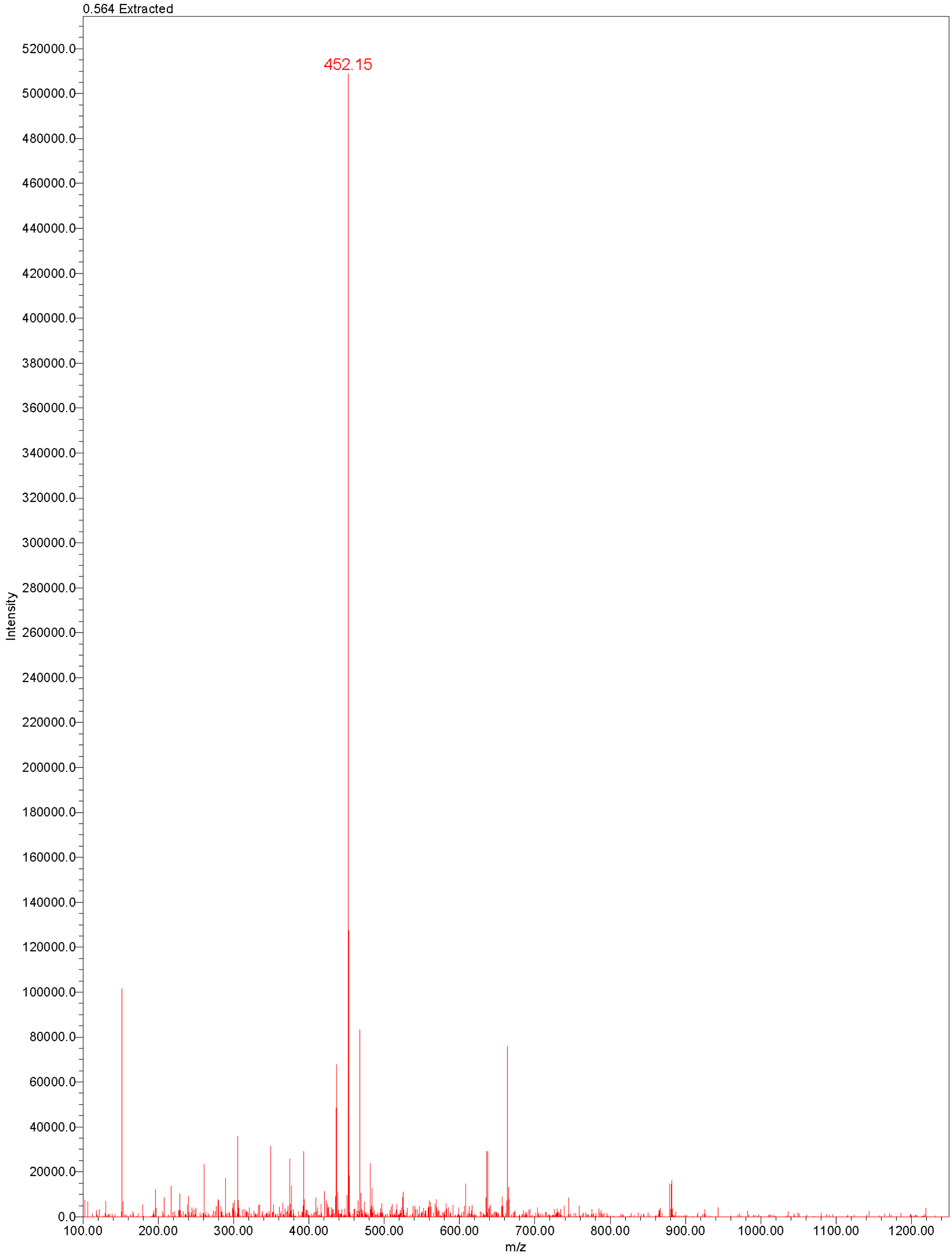

Figure S4. The MS spectrum of Fmoc-diF-Pro-OtBu (**1**): calculated for $[M+Na]^+$ (m/z) of **1** $C_{24}H_{25}F_2NO_4$ 452.2; found 452.2.

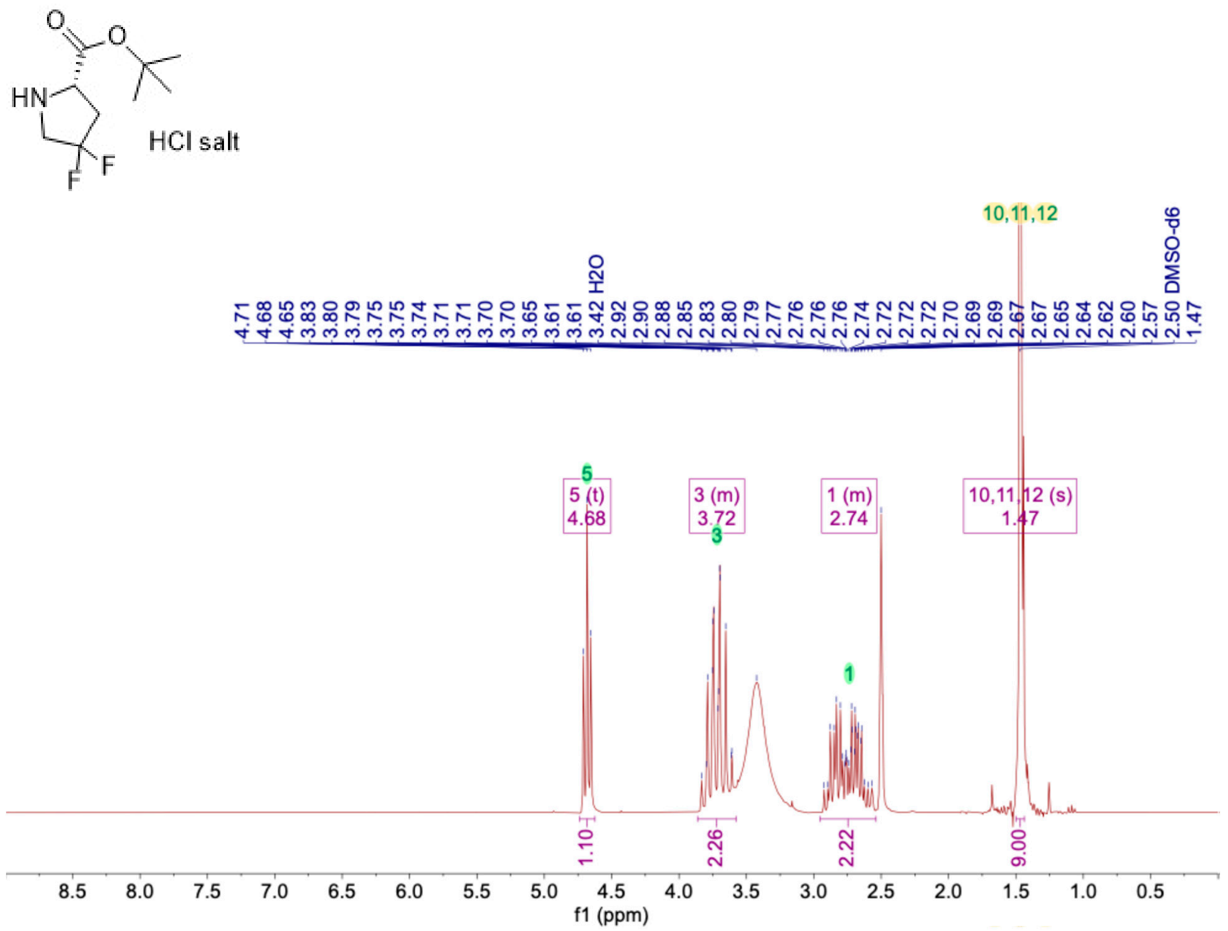

Figure S5. The ^{1}H NMR spectrum of diF-Pro-OtBu HCl salt (**2**).

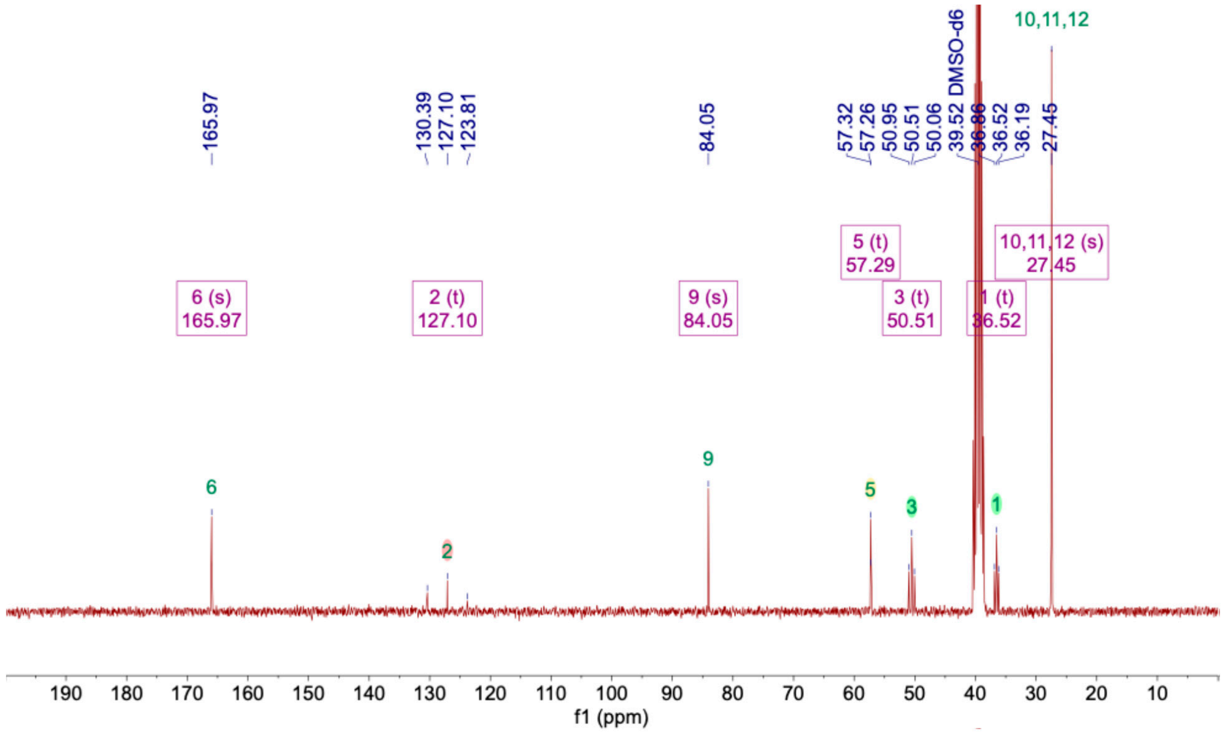

Figure S6. The ^{13}C NMR spectrum of diF-Pro-OtBu HCl salt (**2**).

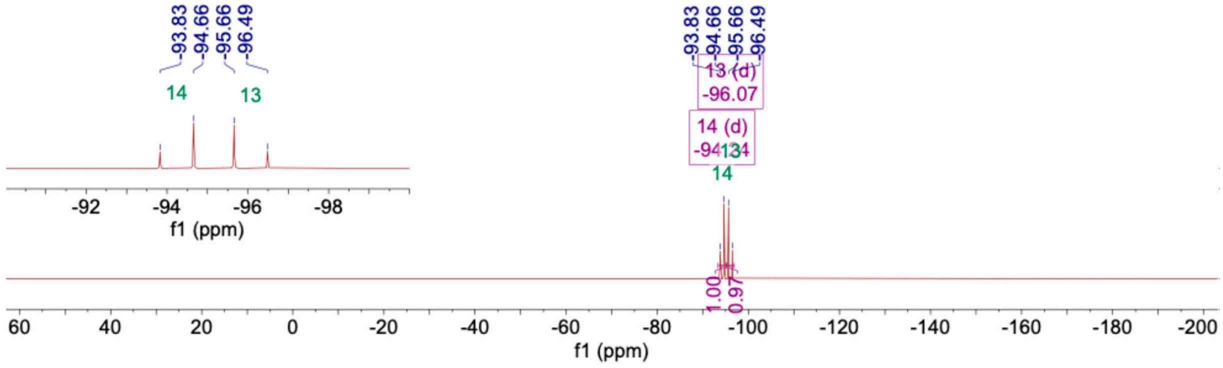

Figure S7. The ^{19}F NMR spectrum of diF-Pro-OtBu HCl salt (**2**).

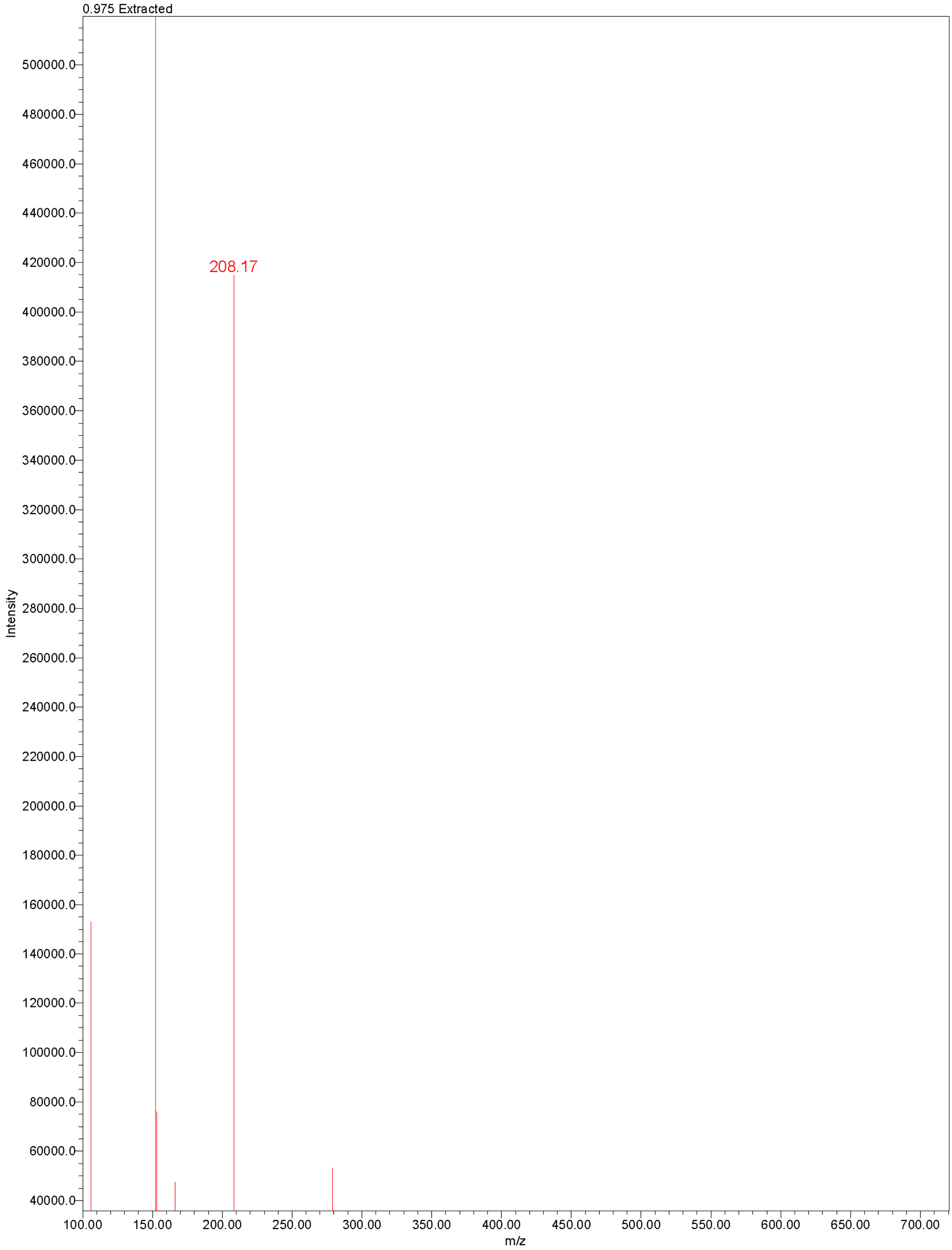

Figure S8. The MS spectrum of diF-Pro-OtBu HCl salt (**2**): calculated for $[M+H]^+$ (m/z) of **2** $C_9H_{15}F_2NO_2$ 208.1; found 208.2.

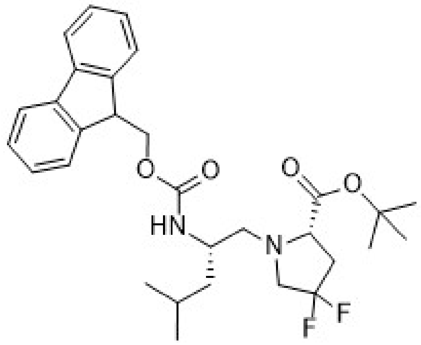

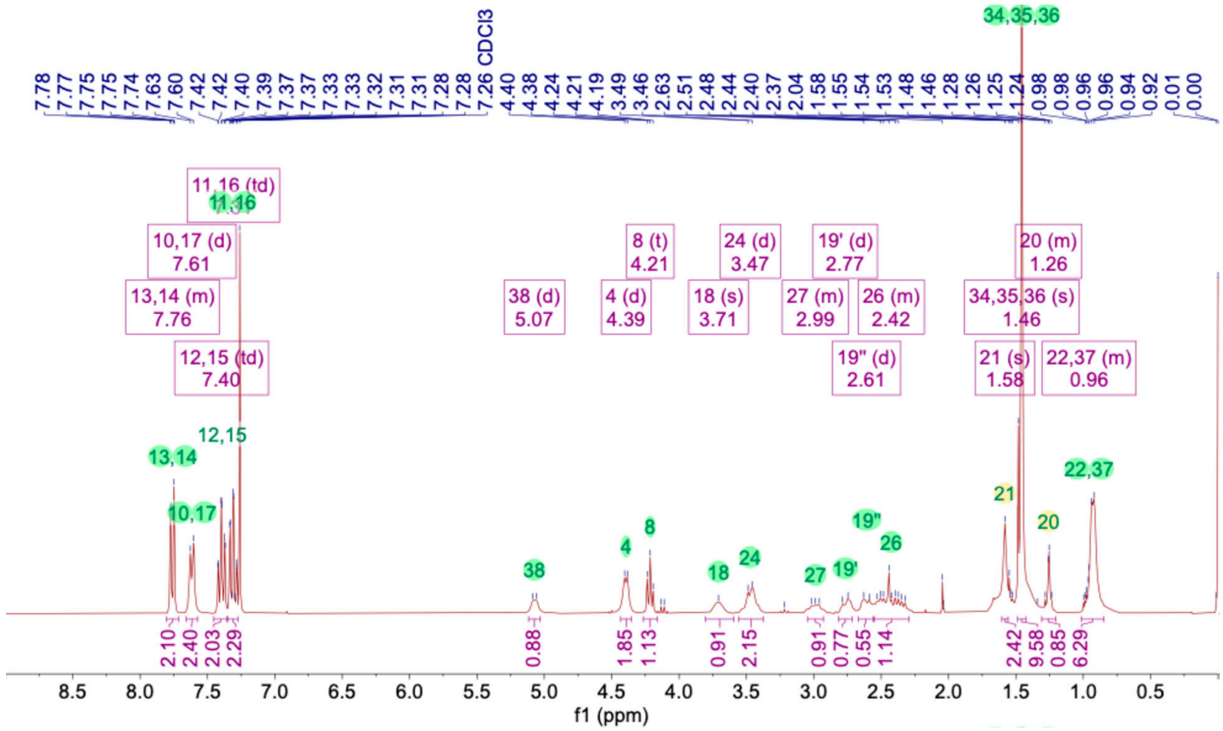

Figure S9. The ^{1}H NMR spectrum of Fmoc-Leu(ψ)diF-Pro-OtBu (**4**).

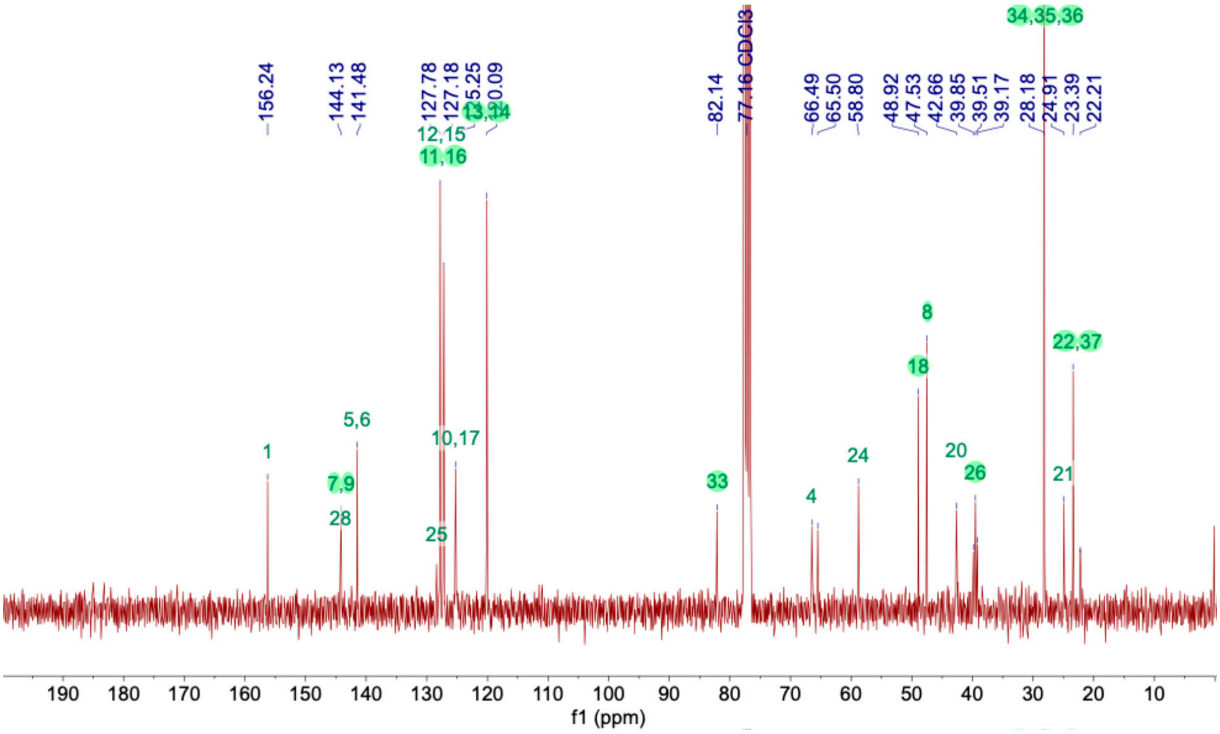

Figure S10. The ^{13}C NMR spectrum of Fmoc-Leu(ψ)diF-Pro-OtBu (**4**).

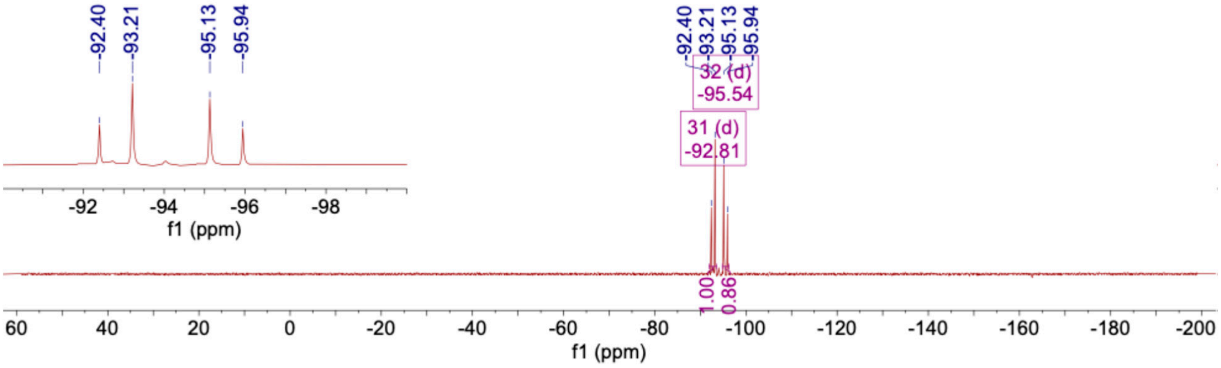

Figure S11. The ^{19}F NMR spectrum of Fmoc-Leu(ψ)diF-Pro-OtBu (**4**).

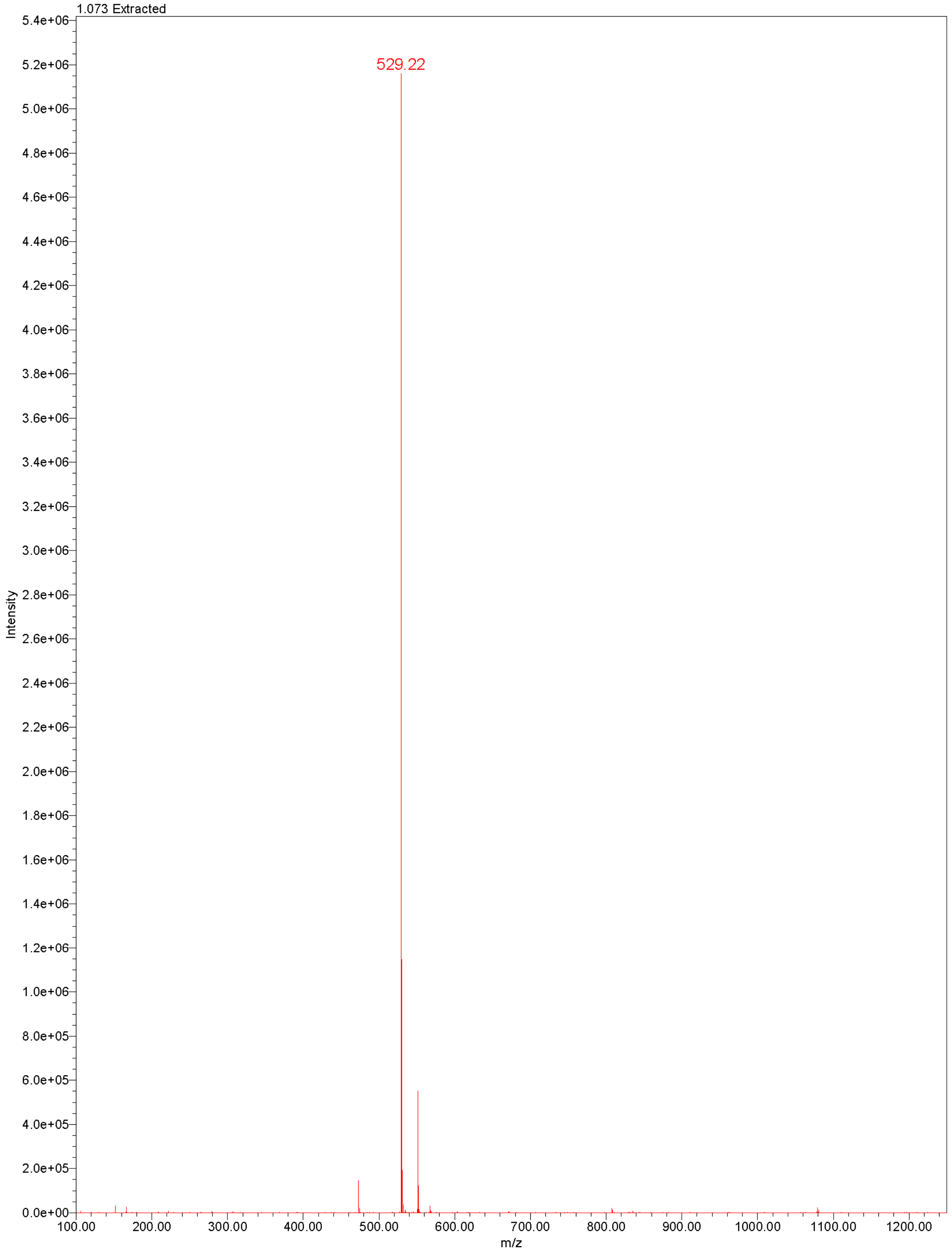

Figure S12. The MS spectrum of Fmoc-Leu(ψ)diF-Pro-OtBu (**4**): calculated for $[M+H]^+$ (m/z) of **4** $C_{30}H_{38}F_2N_2O_4$ 529.3; found 529.2.

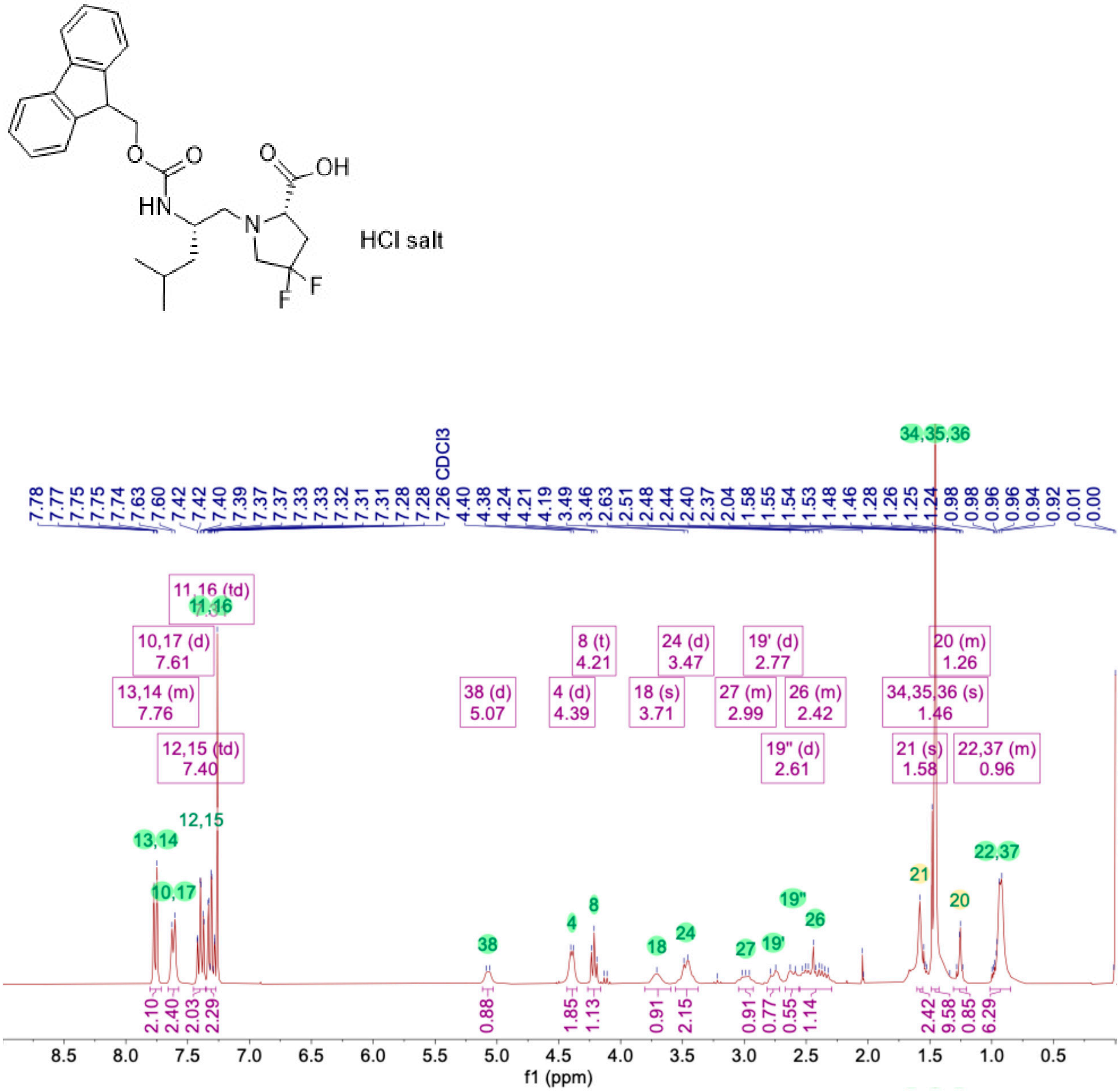

Figure S13. The ^{1}H NMR spectrum of Fmoc-Leu(ψ)diF-Pro-OH HCl salt (**5**).

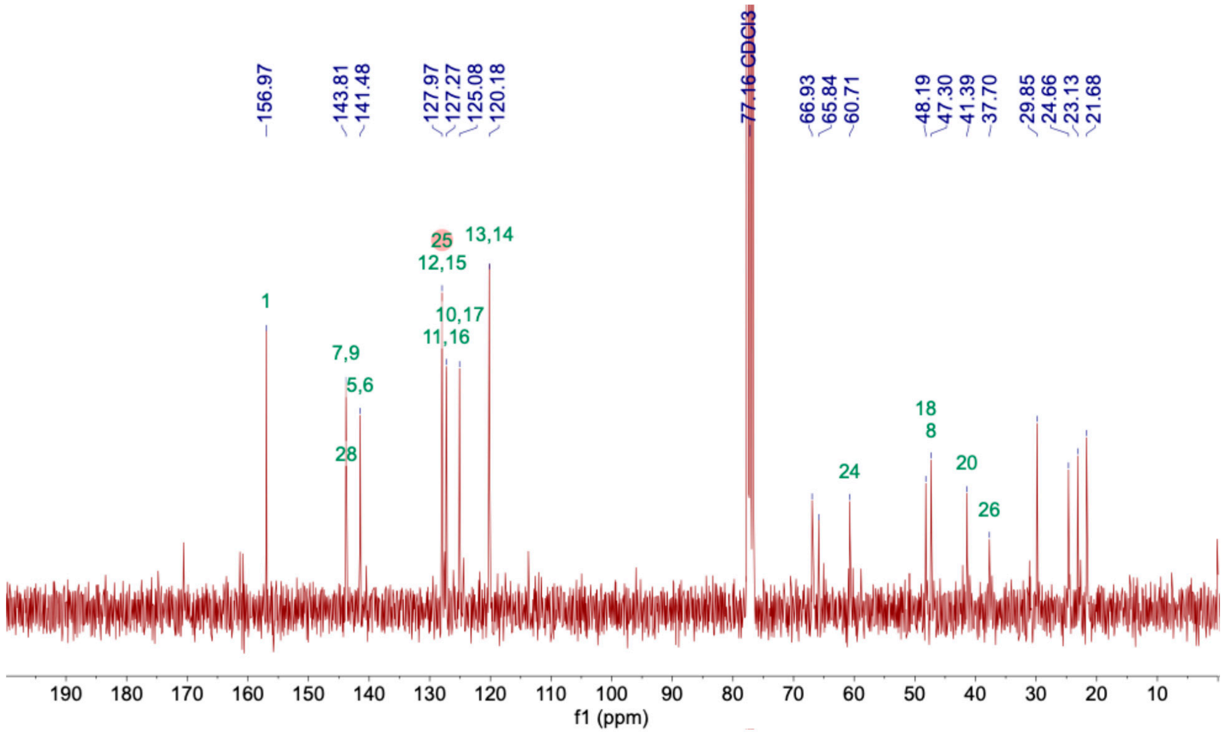

Figure S14. The ^{13}C NMR spectrum of Fmoc-Leu(ψ)diF-Pro-OH HCl salt (**5**).

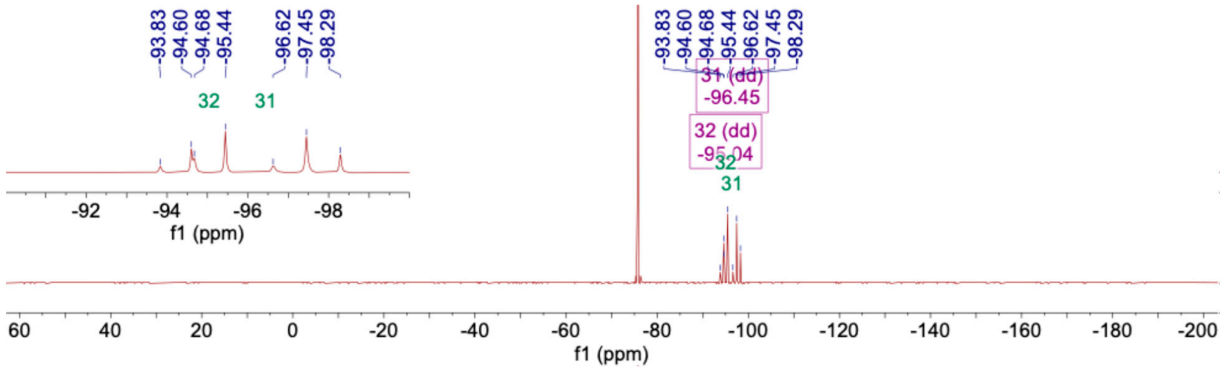

Figure S15. The ^{19}F NMR spectrum of Fmoc-Leu(ψ)diF-Pro-OH HCl salt (**5**).

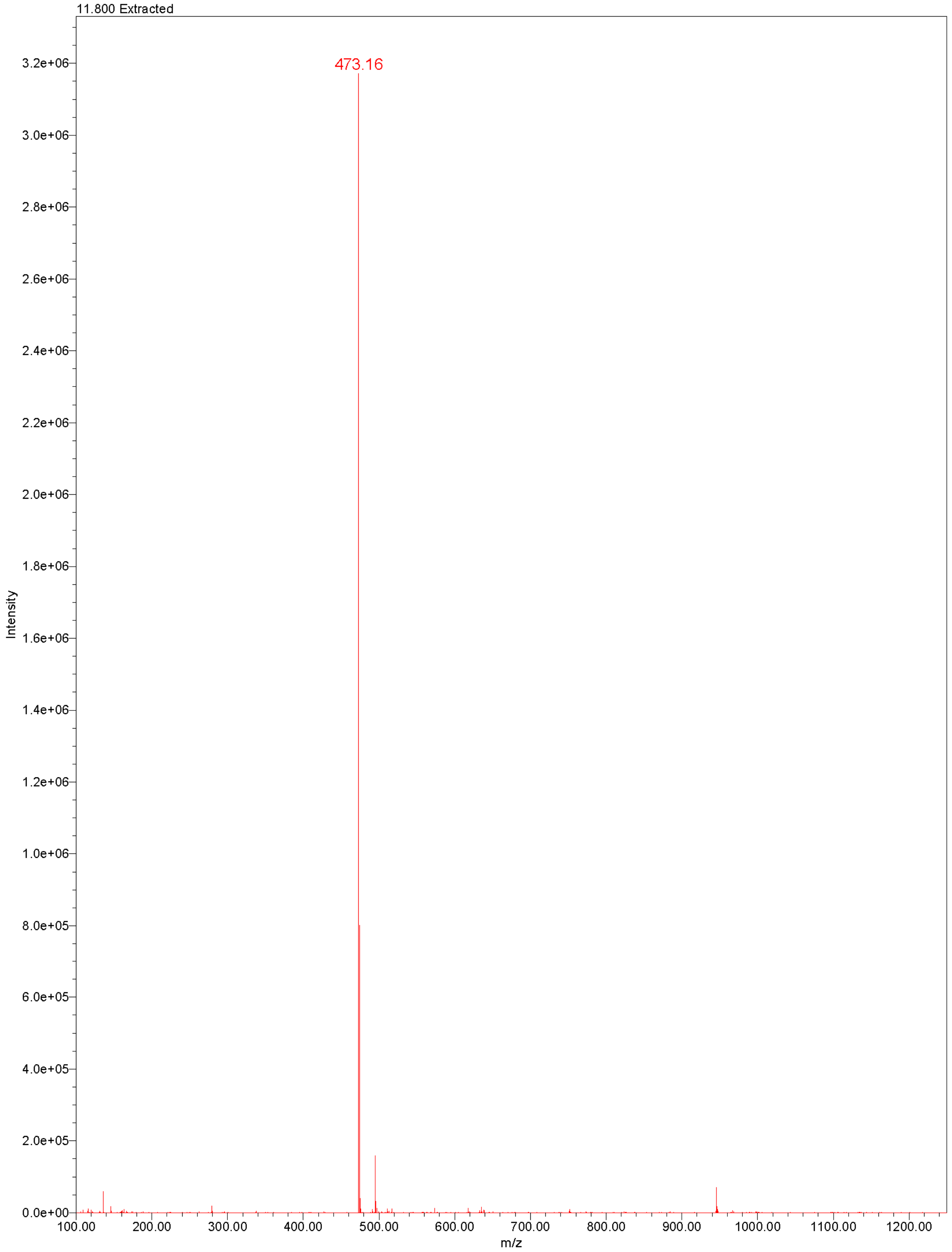

Figure S16. The MS spectrum of Fmoc-Leu(ψ)diF-Pro-OH HCl (**5**): calculated for $[M+H]^+$ (m/z) of **5** $C_{26}H_{30}F_2N_2O_4$ 473.2; found 473.2.

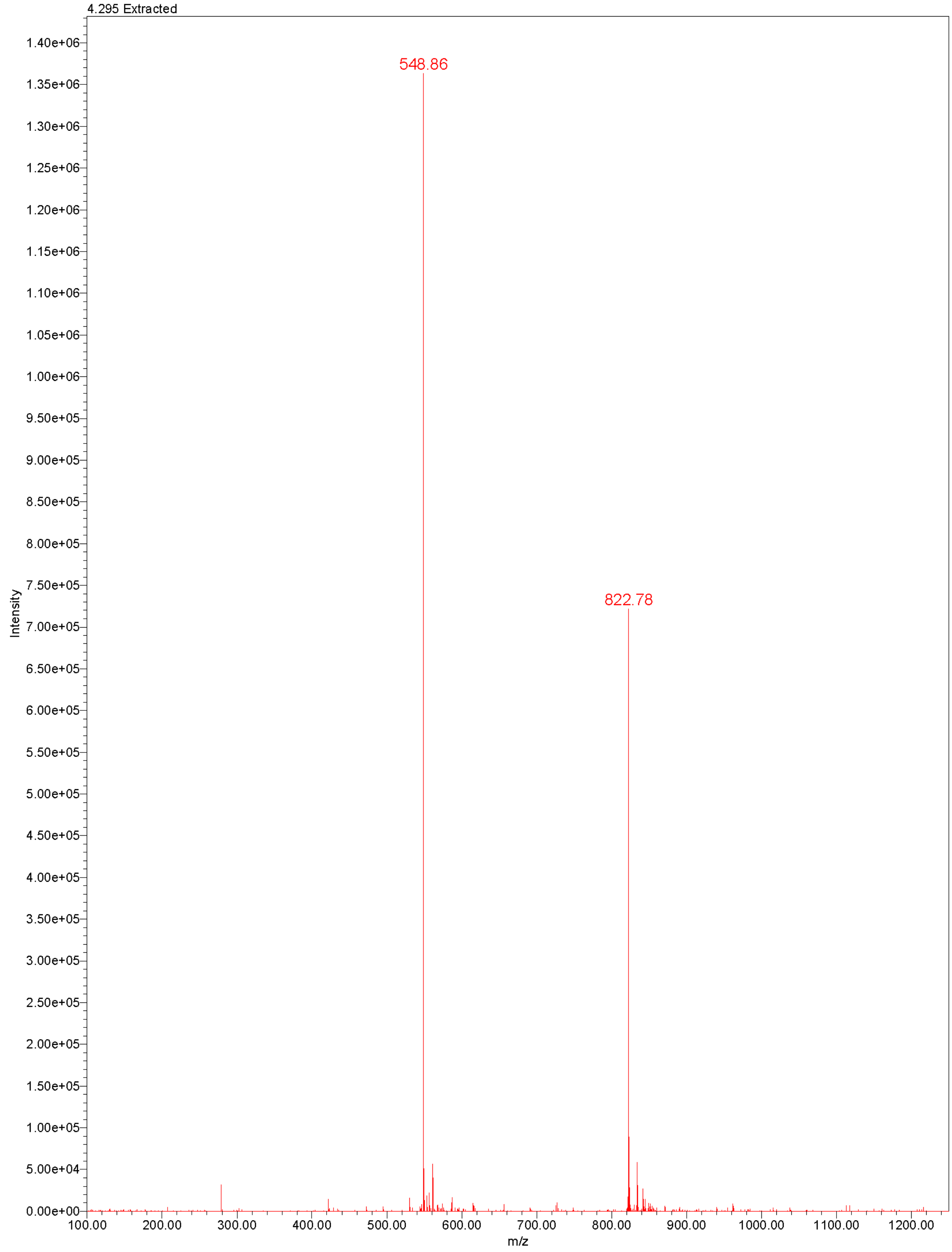

Figure S17. The MS spectrum of LW02060: calculated $[M+2H]^{2+}$ (m/z) 822.4; found 822.8.

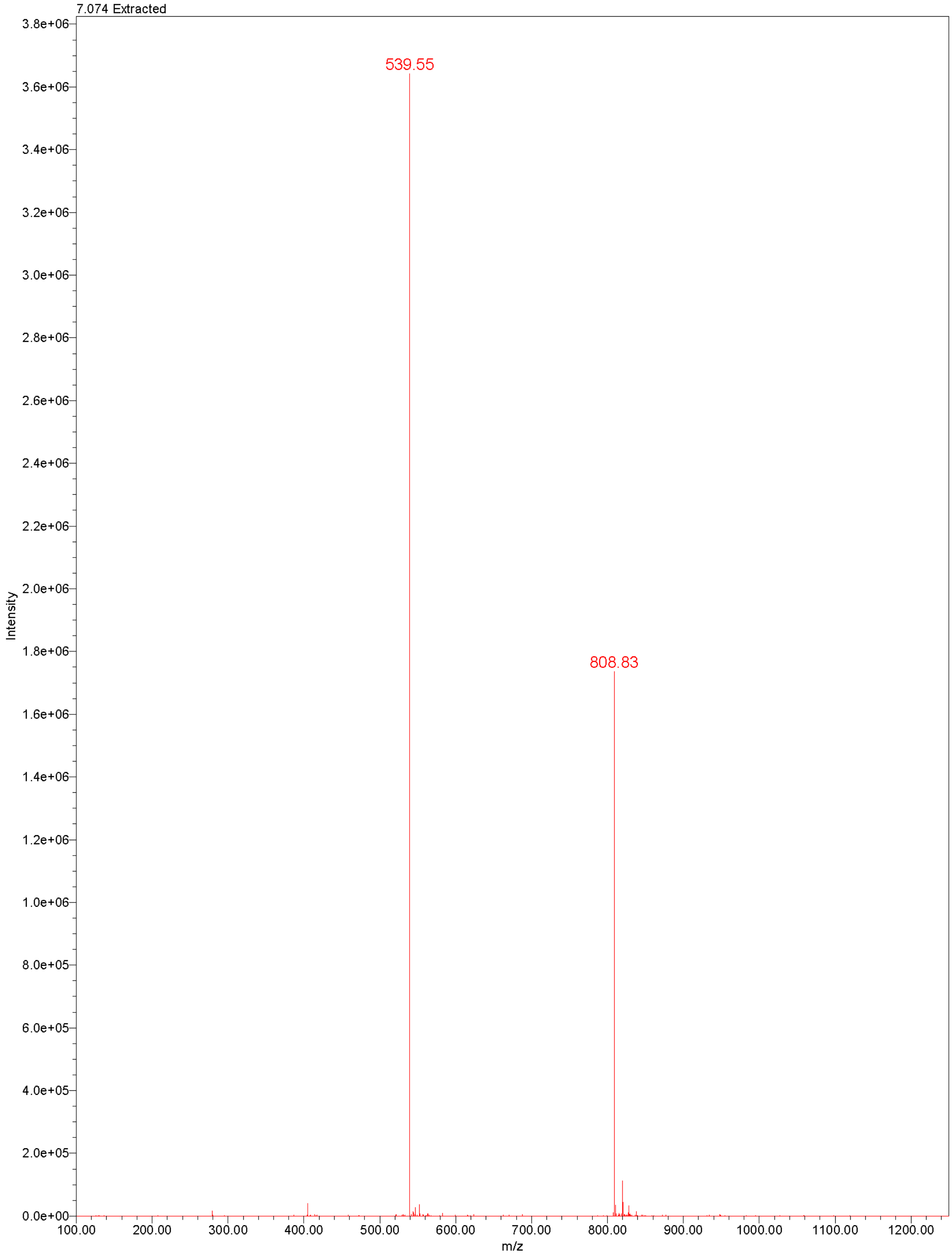

Figure S18. The MS spectrum of LW02080: calculated $[M+2H]^{2+}$ (m/z) 808.4; found 808.8.

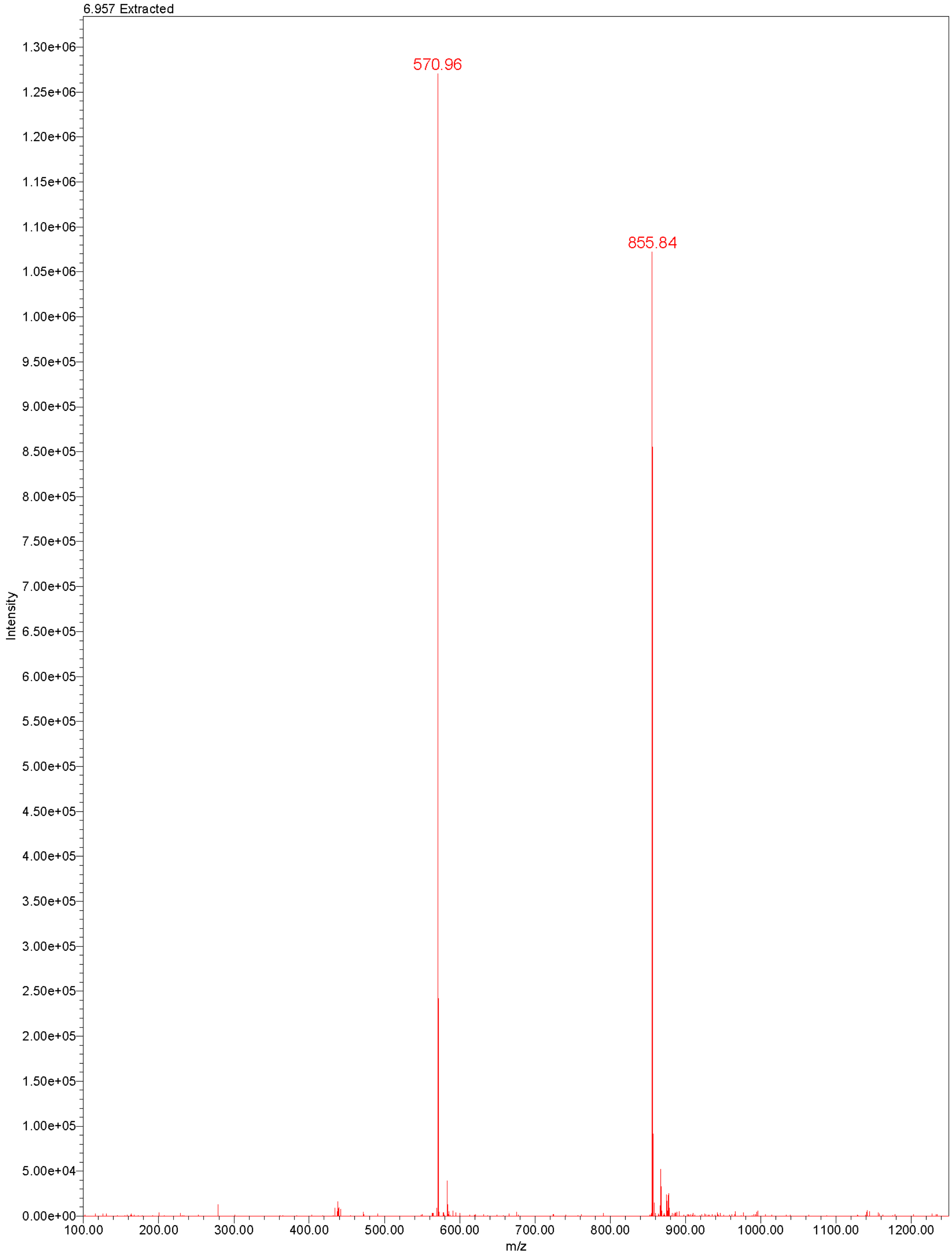

Figure S19. The MS spectrum of Ga-LW02060: calculated $[M+2H]^{2+}$ (m/z) 855.9; found 855.8.

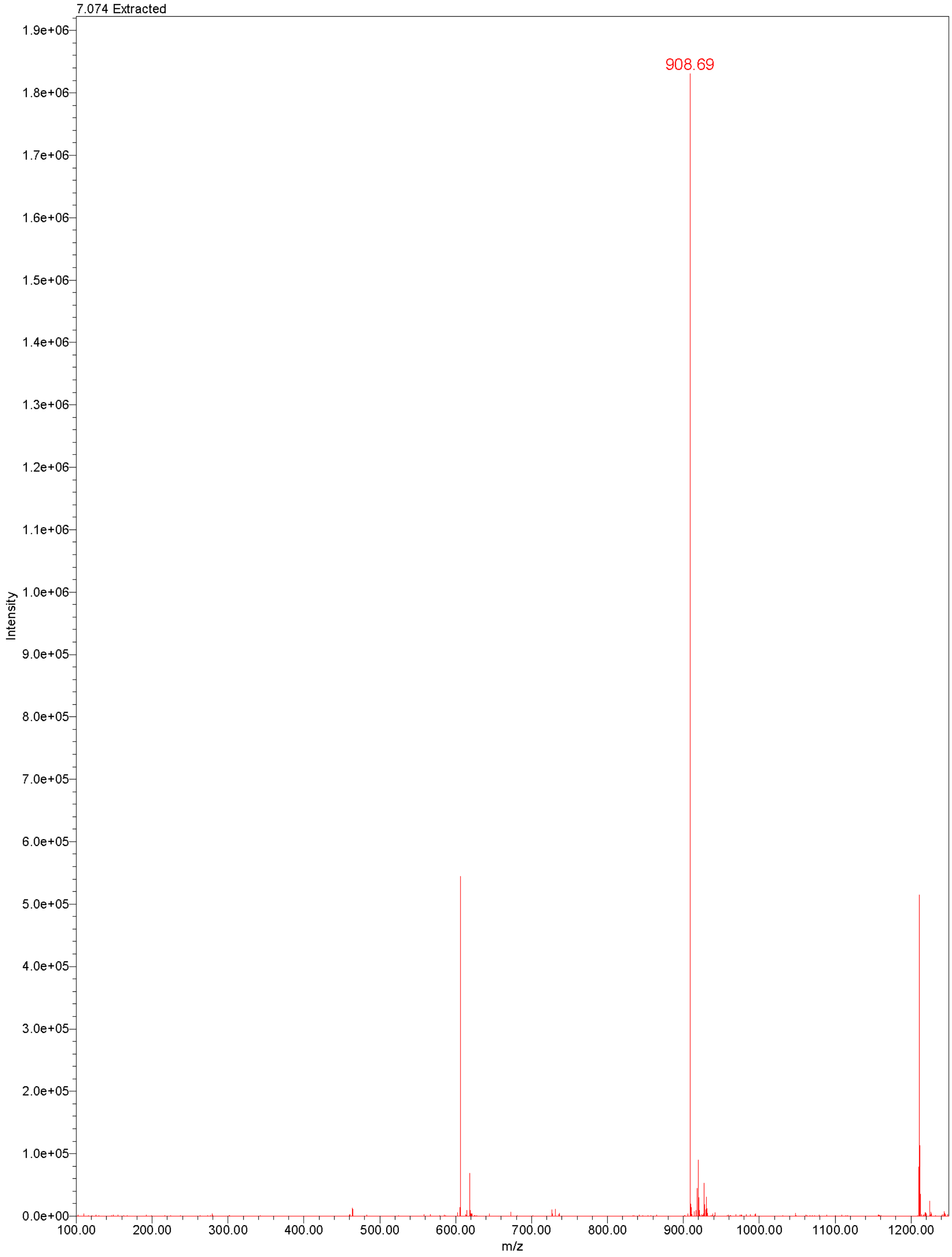

Figure S20. The MS spectrum of Lu-LW02060: calculated $[M+2H]^{2+}$ (m/z) 908.4; found 908.7.

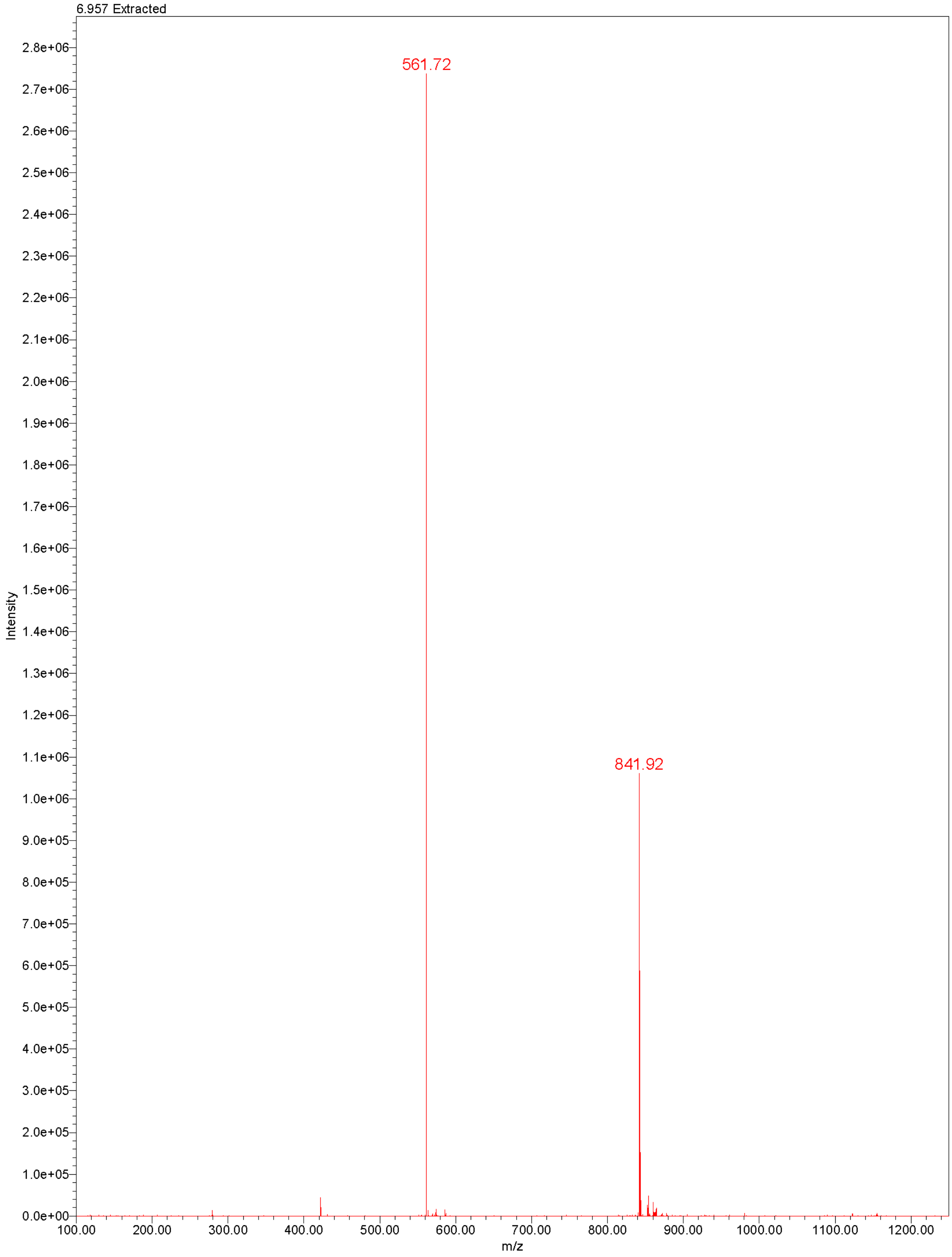

Figure S21. The MS spectrum of Ga-LW02080: calculated $[M+2H]^{2+}$ (m/z) 841.9; found 841.9.

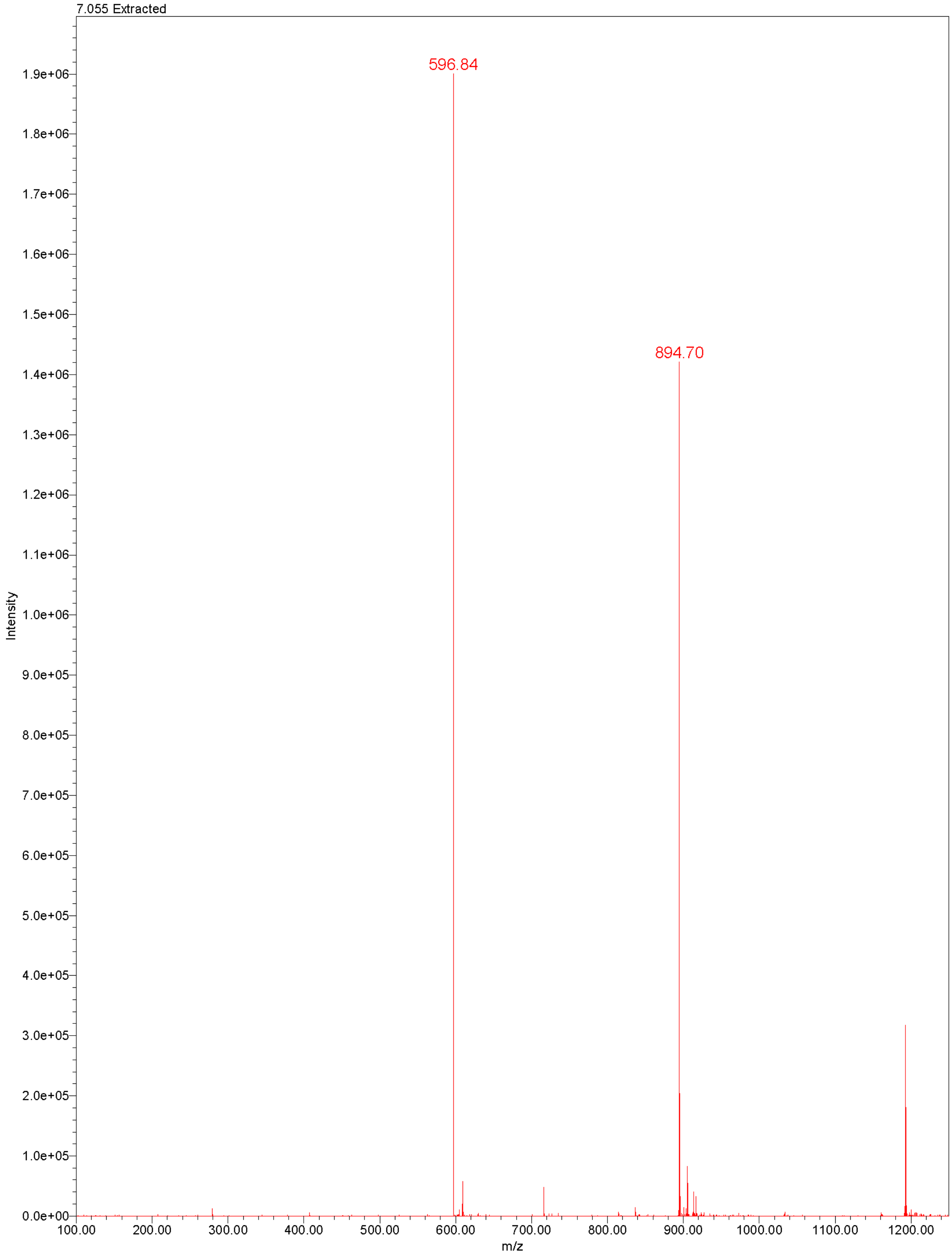

Figure S22. The MS spectrum of Lu-LW02080: calculated $[M+2H]^{2+}$ (m/z) 894.4; found 894.7.

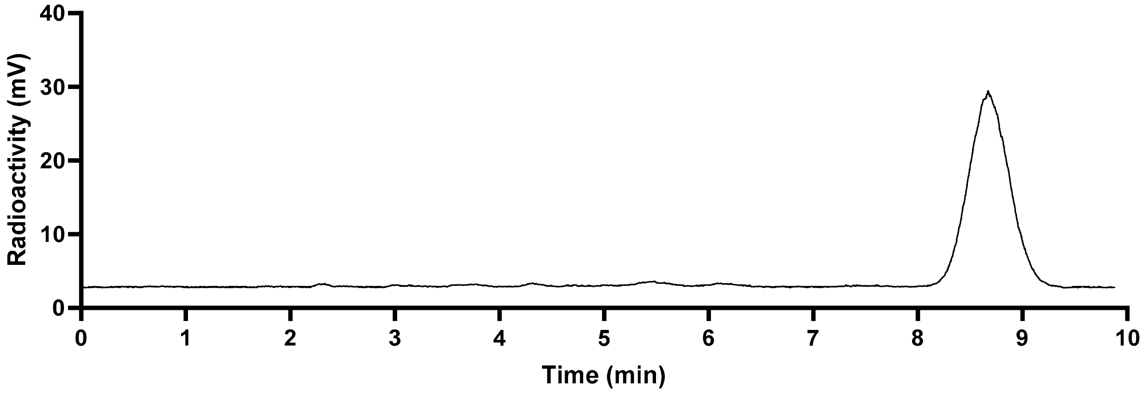

Figure S23. A representative analytical radio-HPLC chromatogram of the purified [^{68}Ga]Ga-LW02060.

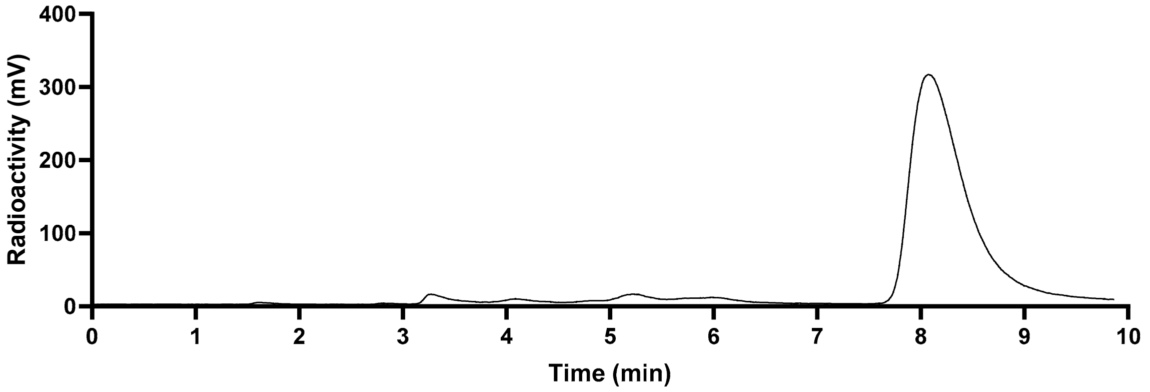

Figure S24. A representative analytical radio-HPLC chromatogram of the purified [^{68}Ga]Ga-LW02080.

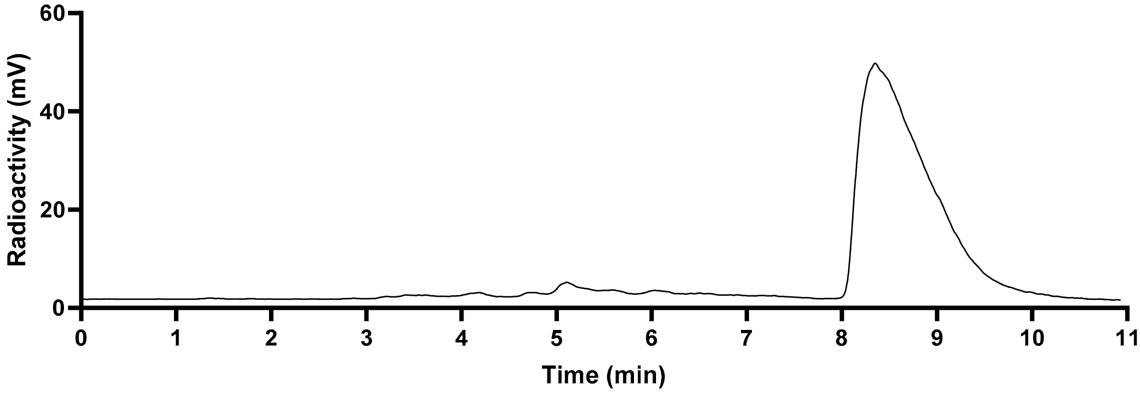

Figure S25. A representative analytical radio-HPLC chromatogram of the purified [^{177}Lu]Lu-LW02060.

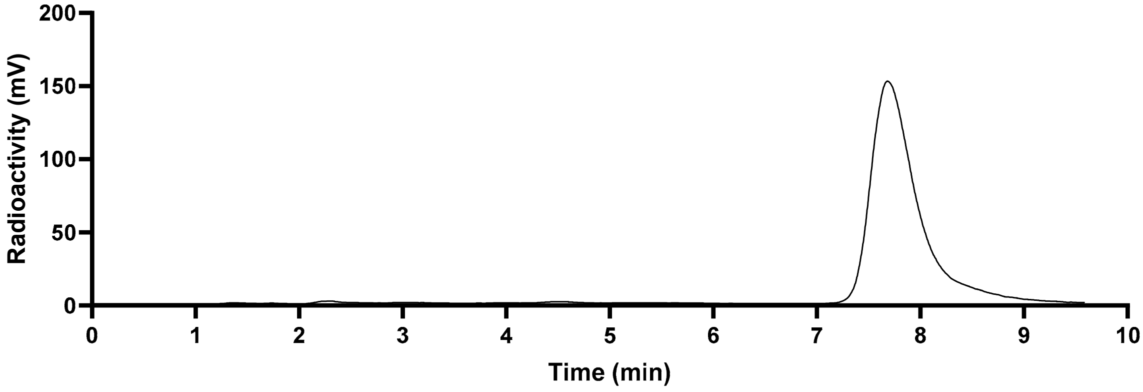

Figure S26. A representative analytical radio-HPLC chromatogram of the purified [^{177}Lu]Lu-LW02080.
